# In silico characterisation of putative *Plasmodium falciparum* vaccine candidates in African malaria populations

**DOI:** 10.1038/s41598-021-95442-4

**Published:** 2021-08-10

**Authors:** O. Ajibola, M. F. Diop, A. Ghansah, L. Amenga-Etego, L. Golassa, T. Apinjoh, M. Randrianarivelojosia, O. Maiga-Ascofare, W. Yavo, M. Bouyou-Akotet, K. M. Oyebola, B. Andagalu, U. D’Alessandro, D. Ishengoma, A. A. Djimde, E. Kamau, A. Amambua-Ngwa

**Affiliations:** 1 Medical Research Council Unit, The Gambia at London School of Hygiene and Tropical Medicine, Banjul, The Gambia; 2 First Technical University, Ibadan, Nigeria; 3 Noguchi Memorial Institute for Medical Research, University of Ghana, P.O. Box LG 581, Legon, Ghana; 4 West African Center for Cell Biology of Infectious Pathogens, University of Ghana, Accra, Ghana; 5 Aklilu Lemma Institute of Pathobiology, Addis Ababa University, Addis Ababa, Ethiopia; 6 Department of Biochemistry and Molecular Biology, University of Buea, Buea, Cameroon; 7 Institut Pasteur of Madagascar, Antanarivo, Madagascar; 8 Bernhard Nocht Institute for Topical Medicine (BNITM), Hamburg, Germany; 9 Unite Des Sciences Pharmaceutiques et Biologiques, University Félix Houphouët-Boigny, Abidjan, Côte d’Ivoire; 10 Faculty of Medicine, University of Health Sciences, Libreville, Gabon; 11 Department of Zoology, University of Lagos, Lagos, Nigeria; 12 United States Army Medical Research Directorate-Africa, Kenya Medical Research Institute/Walter Reed Project, Kisumu, Kenya; 13 National Institute for Medical Research (NIMR), Tanga, Tanzania; 14 Malaria Research and Training Centre, University of Science, Techniques and Technologies of Bamako, Bamako, Mali; 15 Department of Pathology and Laboratory Medicine, David Geffen School of Medicine, University of California, Los Angeles, CA 90095 USA; 16 U.S. Military HIV Research Program, Walter Reed Army Institute of Research, Silver Spring, MD 20910 USA

**Keywords:** Genomics, Sequencing, Infectious diseases, Vaccines

## Abstract

Genetic diversity of surface exposed and stage specific *Plasmodium falciparum* immunogenic proteins pose a major roadblock to developing an effective malaria vaccine with broad and long-lasting immunity. We conducted a prospective genetic analysis of candidate antigens (*msp1, ama1, rh5, eba175, glurp, celtos, csp, lsa3, Pfsea, trap,* conserved *chrom3*, *hyp9*, *hyp10*, *phistb, surfin8.2,* and *surfin14.1*) for malaria vaccine development on 2375 *P. falciparum* sequences from 16 African countries. We described signatures of balancing selection inferred from positive values of Tajima’s D for all antigens across all populations except for *glurp.* This could be as a result of immune selection on these antigens as positive Tajima’s D values mapped to regions with putative immune epitopes. A less diverse *phistb* antigen was characterised with a transmembrane domain, glycophosphatidyl anchors between the N and C- terminals, and surface epitopes that could be targets of immune recognition. This study demonstrates the value of population genetic and immunoinformatic analysis for identifying and characterising new putative vaccine candidates towards improving strain transcending immunity, and vaccine efficacy across all endemic populations.

## Introduction

Malaria remains a deadly disease with major economic implications, imposing hardship and marginalization of poorly resourced communities, especially in sub-Saharan Africa (sSA). An effective malaria vaccine would significantly boost malaria control and elimination efforts. However, genetic diversity and the evolutionary arms race between the parasite and the host drives antigenic diversity of surface proteins, and other life cycle stage-specific proteins targeted as potential vaccine antigens, thereby hampering the development of a widely deployable vaccine^1^.

Field trials of some of the most advanced candidate malaria vaccines have shown strain specificity^2, 3^, raising the possibility of allele-specific immunity as that seen in vaccines against influenza and other diseases^4–7^. Most of the current leading malaria vaccine candidates in clinical trials were designed and developed based on clonal laboratory strains, mostly *Plasmodium falciparum* strain NF54 (clone 3D7), without considering parasite genetic variability in natural populations^8–12^. RTS,S/AS01, the most advanced of these malaria vaccines, is based on a subunit of 3D7 circumsporozoite surface protein (CSP) antigen. It has shown moderate (< 50%) and time-limited protection in African children^13^, partly due to the low prevalence (5.2%) of 3D7-type CSP alleles^9^. Several other recombinant malaria vaccines are currently being evaluated in clinical trials, such as: EBA region II-non-glycosylated (EBA-175 RII NG), apical membrane antigen 1 diversity covering (AMA-1 DiCo), merozoite surface protein-3 (MSP3) long synthetic peptide (LSP), and serine repeat antigen-5. Other recombinant malaria vaccines in clinical trials are described in a recent review by Salamanca et al^10^. Additionally, the Cell-Traversal protein for Ookinetes and Sporozoites (CelTOS), Thrombospondin Related Adhesion Protein (TRAP), and Liver Stage Antigen 1 (LSA1), which target liver stage parasites; merozoite surface proteins (MSP2) which target blood stage parasites; and transmission blocking stage vaccines targeting Pfs230, Pfs45/48, Pfs25 and Pfs48/45^11, 14, 15^ are also in advanced development.

Nevertheless, given our incomplete understanding of the complexity of immune responses to infection, the parasite’s mechanisms of invasion, and the parasite genome^16–19^, most of these vaccine candidates would face most of the challenges observed during the RTS,S/ASO1 development. To overcome some of these difficulties and improve vaccine efficacy by generating cross-protective multi-strain immunity across diverse natural populations, next generation vaccines must use innovative design approaches that account for genetic variables such as differences in antigen allele haplotypes and their frequencies across *P. falciparum* populations^1, 20^.

Exploring the allele frequency spectrum and haplotype diversity of vaccine candidates can inform how natural selection and demographic events shape them across multiple populations. For example, characterising genetic diversity and signatures of selection in the parasite genome or for specific antigens has been useful in identifying genes under balancing selection and genic regions that are immunogenic for further development^20–22^. With the increase of next generation sequencing and openly accessible genome data from multiple endemic populations, future multi-allelic vaccines can benefit from genome scans for signatures of balancing selection. This will allow assessment of allele and haplotype frequencies across a broad range of endemic populations, particularly in sSA where parasite genetic diversity is high^22^. Such in silico diversity and prediction approaches should include sequence data obtained across multiple temporal and spatial populations. This could be explored to identify new candidates, refine components of current candidate vaccine antigens, with optimal components of relevant alleles from both pre-erythrocytic and erythrocytic targets prior to functional characterisation and clinical trials^15^.

Here, we conducted population genetic and immunoinformatic analysis of selected malaria candidate vaccine antigens using single nucleotide polymorphisms (SNP) from field isolates from 16 African countries. We estimated the extent of genetic diversity in these antigens and evaluated the evidence of balancing selection that could be shaping their evolution. From this large population dataset, we described a novel putative candidate vaccine antigen—*phistb*—that could be explored for monovalent vaccine development or as part of a multivalent vaccine. Together, this is the largest exploration of candidate vaccines’ genetic diversity for natural populations of malaria parasites from sSA, providing a framework for designing future functional studies and effective malaria vaccines.

## Methods

### Ethical considerations

This study utilized data previously published by the Plasmodium Diversity Network Africa (PDNA)^23, 24^ and the open-source, MalariaGEN (https://www.malariagen.net/projects/pf3k). For the PDNA data, field studies were approved by the respective local ethical review committees. Venous blood (2–5 ml) was collected from study participants aged > 6 months enrolled under approved protocol(s) in each country. All participants and/or their legal guardians provided written informed consent before any study procedures. All procedures performed in studies involving human participants were in accordance with the Declaration of Helsinki. Samples were leukocyte depleted and extracted DNA sequenced by MalariaGEN at the Wellcome Sanger Institute.

To utilize PDNA and the open-source data in the current analysis, this study was elaborated as part of a malaria genomic surveillance proposal reviewed and approved by the Gambian Government/MRC Joint Ethics Committee. The methods employed in this study were in agreement with the MRCG-LSHTM research governance policy.

### Vaccine candidates

A total of 16 *Plasmodium falciparum* vaccine antigens were identified and analysed; among them, 10 (*msp1, ama1, rh5, eba175, glurp, celtos, csp, lsa3, Pfsea* and *trap*) were included as potential vaccine antigens. The remaining 6 antigens (conserved *chrom3*, *hyp9*, *hyp10*, *phist,* and *surfin8.2, surfin14.1*) were identified using Rsb (a metric for comparing integrated extended haplotype homozygosity between populations), the cross population test for signatures of selection^23^. Antigens were classified as pre-erythrocytic (*celtos, csp, lsa3, Pfsea* and *trap*) or erythrocytic (*msp1, ama1, rh5, eba175, glurp, chrom3*, *hyp9*, *hyp10*, *phistb, surfin8.2* and *surfin14.1*) candidates based on the *P. falciparum* life cycle stage of expression.

### Sequences

The *P. falciparum* antigen sequences (2375) used in this study were obtained from PDNA study sites from; Gambia (n = 247), Mali (n = 137), Cote-d’Ivoire (n = 70), Ghana (n = 423), Nigeria (n = 34), Cameroon (n = 239), Gabon (n = 55), Kenya (n = 64), Tanzania (n = 300), Ethiopia (n = 34) and Madagascar (n = 25)^23^. Additionally, open-source sequence data from Guinea (n = 143), Senegal (n = 137), Democratic Republic of Congo (n = 112), Mauritania (n = 86) and Malawi (n = 269) (Fig. S1) were accessed via the Pf3K project (https://www.malariagen.net/projects/pf3k). Details of the methods and curated genotype data can be accessed from https://www.malariagen.net/resource/26 and associated publications^25^.

### Data analysis

#### Nucleotide diversity analysis and neutrality test

Population genetic analyses were carried out on each candidate vaccine dataset to investigate the genetic diversity as well as the frequency of known haplotypes being incorporated into vaccine candidates. The range and distribution of genetic diversity within and among the natural *P. falciparum* populations from individual countries were determined. Population genetic parameters were determined using PopGenome R package^26^. Nucleotide diversity (π) (average number of nucleotide differences per site in pairwise comparisons among DNA sequences) and theta (Ɵ) (population mutation rate) was measured for each candidate vaccine antigen by country. Nucleotide diversity was calculated both for the entire coding sequence of each antigen and in sliding windows to determine regions of increased diversity. Haplotype diversity (Hd) which represents the probability that two randomly sampled alleles are different was estimated per antigen and population.

Neutrality tests designed to distinguish between neutrally evolving sequences under mutation-drift equilibrium- population expansion and contraction, and sequences evolving under non-neutral processes including directional or balancing selection, were derived. We determined Tajima’s D, Fu & Li’s F* and D* statistics. Tajima’s D uses the frequency of segregating nucleotide sites, while Fu’s F* uses the distribution of alleles or haplotypes. Both tests are based on the principle that a rapid population expansion associated with a non-neutral process will show a shift in the allele frequency spectrum compared to a neutral Wright-Fisher model consistent with population expansion under neutral evolution. For all the candidate vaccine antigens, Tajima’s D was estimated by country using vcftools^27^ for the entire antigen region or in consecutive sliding windows of 100 bases. Positive Tajima’s D values generally suggest balancing selection, or a population contraction is acting to maintain alleles at intermediate frequencies; negative values suggest purifying or positive selection, or population expansion that results in an excess of rare alleles.

#### Linkage disequilibrium (LD)

To determine correlation between antigen alleles, pair-wise LD between different polymorphic sites was computed based on the genotype correlation coefficient (r^2^) index between alleles at physically separate loci with a minor allele frequency > 1% across all populations using PLINK v1.90b6.4^28^. Within each population, r^2^ measures for each antigen were calculated between all pairs of single nucleotide polymorphisms (SNP) and observed pair-wise LD (r^2^) was averaged for each inter-SNP distance. Decay of LD with physical distance between SNP loci was fitted in R version 3.6.

#### Population differentiation among sampling populations

To assess geneflow between populations, we first estimated genetic differentiation (i.e. the difference in the average diversity within populations compared to that among populations) expressed as Wright’s fixation index using whole SNP dataset for each vaccine candidate^29^. The fixation index (*F*_ST_) was estimated for pairs of populations using the hierFstat package^30^. For interpretation, *F*_ST_ values < 0.05 was delineated as low differentiation or high gene flow between population pairs, 0.05–0.15 as intermediate, and > 0.25 as high differentiation.

#### Haplotype cluster network analysis

Genetic relationships between antigen haplotypes of *P. falciparum* from the 16 African countries were constructed using the median joining algorithm in the R packages Pegas and Ape^31^. The haplotype network was computed using haplotype pairwise differences as a distance measure, estimated from the number of SNP variants between haplotypes.

#### B-cell epitope prediction for vaccine candidates

The random forest algorithm trained on epitopes annotated from antibody-antigen protein structures was employed in predicting linear B-cell epitopes for 11 candidate vaccine antigens with at least 3 SNPs, by employing BepiPred-2.0 at a threshold of 0.5^32^. Sequences of these antigens were uploaded on to the server in FASTA format and B-cell epitopes returned as outputs. We further compared the B cell epitopes predicted with segments of the antigens’ sequences with elevated positive Tajima’s D values. This allowed us to map contiguous regions in the proteins which are likely under immune selection and could be included in future designs of multivalent malaria vaccines.

#### three-dimensional structure and membrane anchor prediction for Phistb

The amino acid sequence of the candidate vaccine antigen was submitted to I-TASSER for in silico prediction analysis using the default settings^33^. The predicted model with the highest C-score (range: 0–1), used for indicating confidence in the predicted models, was selected and epitopes and regions under balancing selection highlighted. Transmembrane helices and glycophosphatidylinositol (GPI) anchors were predicted by uploading *phistb* sequence into the TMHMM prediction servers and GPI-SOM respectively^34^.

### Statistical analyses

Spearman's rank nonparametric correlation coefficient was used to measure the correlation between the three neutrality tests applied. Mann–Whitney *U* non-parametric test was used to assess statistically significant differences between groups assuming a non-Gaussian distribution. Statistical analysis was performed using GraphPad Prism version 8.0. Additionally, statistical comparison of genetic indices for antigens based on region of origin following stratification of populations into two geographical areas: West and Central Africa (Cameroon, Congo, Cote d’Ivoire, Gabon, Gambia, Ghana, Guinea, Mali, Mauritania, Nigeria, and Senegal) versus East Africa (Ethiopia, Kenya, Madagascar, Malawi and Tanzania). This stratification was based on previous reports by the PDNA describing the clustering of African malaria parasites into subpopulations^23^.

## Results

### Study population and candidate vaccine antigens

The population dataset used in this study comprise 2375 sequences from West, Central, South Central, East and South-Eastern African countries. Three countries in the population dataset; Nigeria, Ethiopia and Madagascar had fewer parasite sequences (34, 34, and 25 respectively) compared to other countries in our analysis.

### Polymorphism and haplotype diversity

The lowest median number of haplotypes per country was 3 for *pfsea*, and *msp1,* while the highest was 110 for *surfin8.2* (Table S1). All candidate vaccine antigens were initially subjected to nucleotide diversity and F_ST_ analyses. The average pairwise nucleotide difference per site estimated as π and Ɵ for the pre-erythrocytic (*celtos, csp, lsa3* and *trap*) and erythrocytic (*msp1, ama1, rh5, eba175, glurp, chrom3*, *hyp9*, *hyp10*, *phistb, Pfsea, surfin8.2* and *surfin14.1*) antigens showed large nucleotide diversity when aggregated across all countries. For the pre-erythrocytic antigens, *pfsea* had consistently lower π and Ɵ values of 0.00002890 and 0.00009980 respectively (Table 1). The erythrocytic antigens *hyp9* had the lowest diversity values (π = 2.538 × 10^−4^; θ = 8.066 × 10^−4^) whilst *hyp10* had the highest (π = 0.016099; θ = 0.011532)(Table 1). In general, pre-erythrocytic antigens were less diverse than erythrocytic antigens. Nevertheless, when disaggregated by country, the π for pre-erythrocytic antigens ranged from as high 0.0175342 for *csp* (Gabon) to as low as 0.0000075 for *pfsea* (Mauritania). For the erythrocytic antigens, the π value was as high as 0.0189404 for *hyp10* (Gambia) while the lowest values was 0.0000952 for *hyp9* (Cote d’Ivoire), (Table S1). Across all countries, we observed relatively higher genetic diversity in *csp* (except for Ethiopia) and *hyp10.* Nevertheless*,* pre-erythrocytic antigens *lsa3* and *pfsea* had low (0.058–0.096) nucleotide diversity across all countries. Such low levels of diversity were also observed across geographical sites for erythrocytic antigens *rh5, hyp9,* and *glurp* (majority of sites). Interestingly, we observed a relatively higher nucleotide diversity at *rh5* in east African sites (Kenya, Malawi and Tanzania) and the island of Madagascar (Table S1).

**Table 1:** Haplotype diversity of *P. falciparum* candidate vaccine antigens.

Vaccine candidates	Pi(π)	Hd	Theta (Ɵ)
**Pre-erythrocytic**			
celtos	0.00165175	0.9860	0.00165175
csp	0.01555860	0.9490	0.01219290
lsa3	0.00059395	0.8705	0.00049910
trap	0.00828865	0.9965	0.00537735
pfsea	0.00002890	0.0990	0.00009980
**Erythrocytic**			
ama1	0.00165175	0.9955	0.00447465
chrom3	0.00483740	0.6690	0.00363820
eba175	0.00181675	0.9930	0.00105945
glurp	0.00099680	0.9555	0.00151625
hyp9	0.00025385	0.0580	0.00080655
hyp10	0.01609915	0.7995	0.01153165
msp1	0.00306515	0.9985	0.00300525
phistb	0.00160080	0.8335	0.00122970
rh5	0.00087060	0.5530	0.00095175
surfin8.2	0.00808670	0.9990	0.00413205
surfin14.1	0.00153700	0.9965	0.00091120

*Pi* and *theta* nucleotide diversity indexes and *hd,* haplotype diversity.

The haplotype diversity index *(Hd)* values across all antigens and populations were mostly > 0.4 with the exception of pre-erythrocytic antigen *pfsea,* (*Hd* ranging from 0.05 to 0.29) and erythrocytic antigen *hyp9* (0.017–0.153) which were low across all populations (Table S1). Candidate vaccine antigens *ama1*, *eba175* and *glurp* from Ethiopia had lower *Hd* values than in the other African countries. A similar pattern was observed for pre-erythrocytic antigens. However, it is important to note that a small number of samples were analysed from Ethiopia. To identify any differences within Africa, we compared *Hd* values from the two subregions, namely West and Central Africa versus East Africa, and found significant differences for *lsa3* (*P* = 0.01), *hyp10* (*P* = 0.0005), *phistb* (*P* = 0.0005) and *rh5* (*P* = 0.0032). In our subsequent analysis, antigens with limited SNP information were excluded, which led to removal of five antigens (*pfsea, hyp9, hyp10*, *conserved chrom3* and *rh5*) from Tajima’s D and LD analysis. The remaining 11 antigens were subjected to Tajima’s D, LD and linear B-cell epitope and 3D-structure prediction.

### Genetic differentiation and gene flow

**Figure 1:**
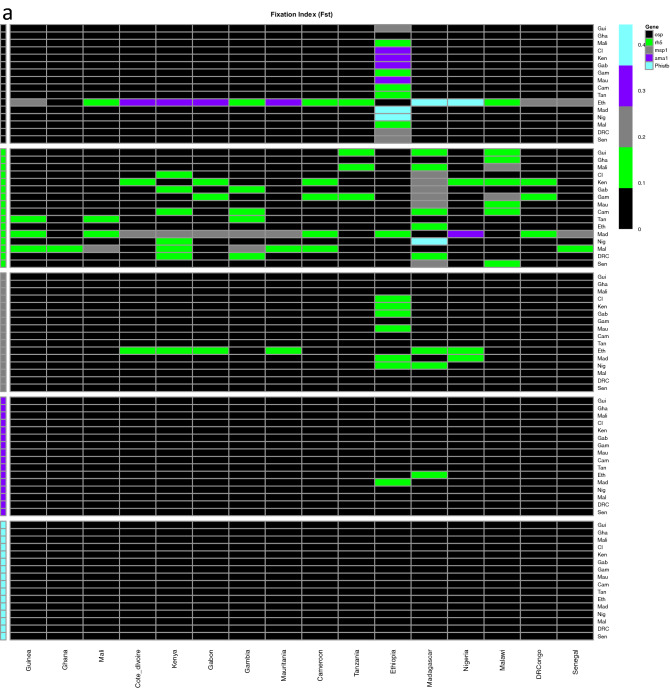

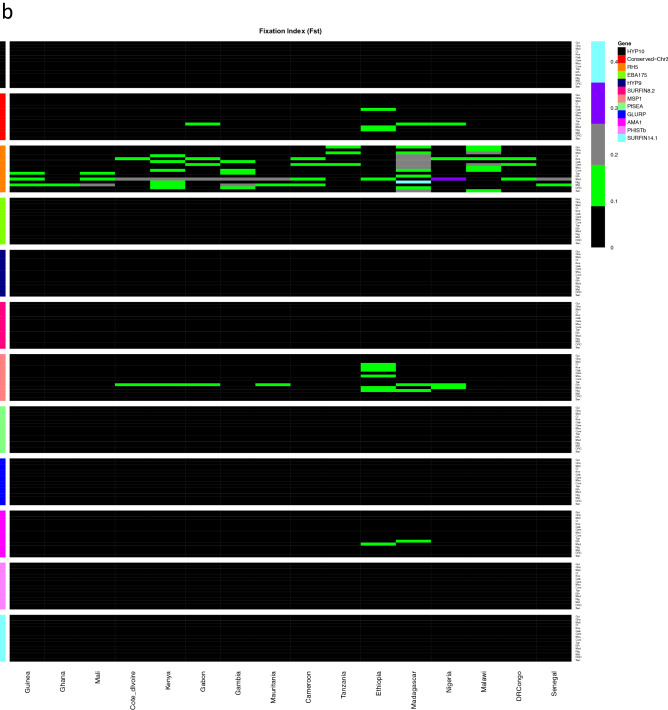
Pairwise F_ST_ indices of candidate vaccine antigens from the 16 African countries, (**a**) Pre-erythrocytic antigens (**b**) erythrocytic antigens. Fst indices indicate low genetic differentiation for most of the antigens (F_ST_ < 0.05). Ethiopia and Madagascar had few samples, 34 and 25 respectively.

We evaluated genetic differentiation among parasite populations to obtain an insight into gene flow by calculating Wright’s fixation index (*F*_ST_) of inter-population variance in allele frequencies. Pairwise *F*_ST_ values for the pre-erythrocytic antigens were low suggesting low genetic differentiation among the populations or extensive genetic exchange (high gene flow), with the exception of *csp* in populations with low samples sizes; Nigeria, Ethiopia and Madagascar, which had high values, *F*_ST_ > 0.25 (Fig. 1). The highest pairwise *F*_ST_ value between Ethiopia and Nigeria for *csp* was (*F*_ST_ = 0.44). Pairwise *F*_ST_ values for erythrocytic antigens also showed low genetic differentiation, with the exception of *rh5* antigen between Madagascar and Gambia (*F*_ST_ = 0.3), which are separated by the widest geographic distance.

### Genetic polymorphisms and haplotype frequency

**Figure 2:**
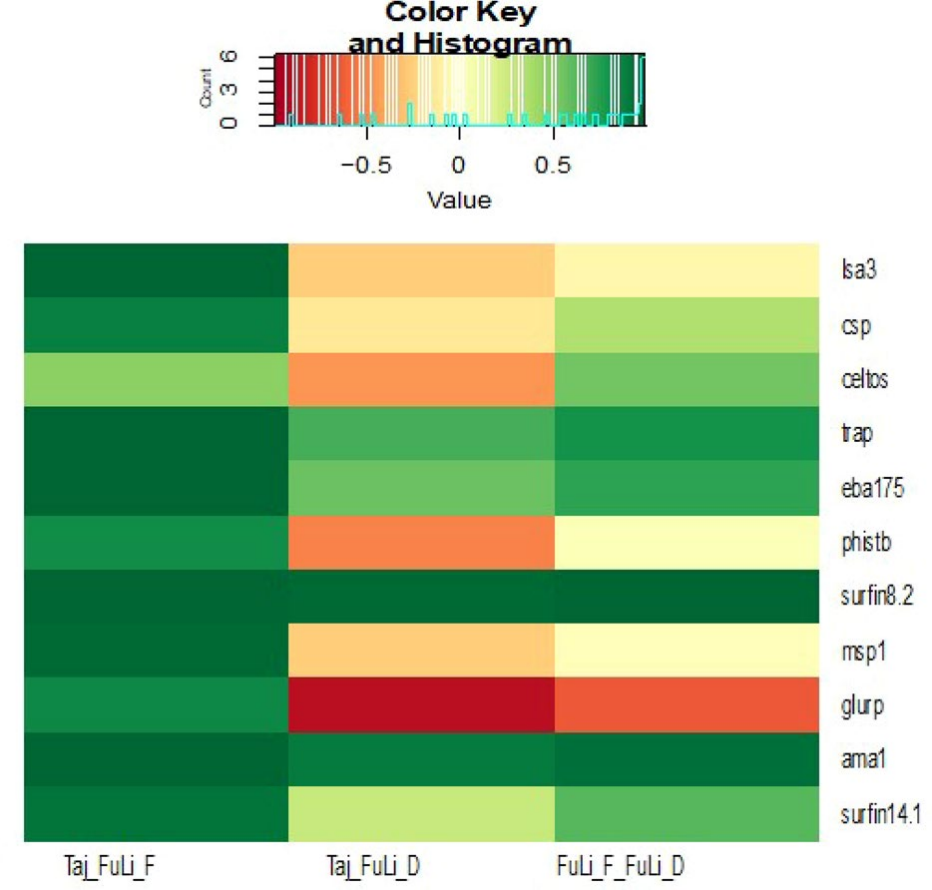
Relationship between Tajima’s D, Fu_Li D* and Fu_LiF*. Positive correlation is indicated by light green to deep green, while negative correlation is represented by light yellow to red.

Across all the neutrality tests (Tajima’s D, Fu & Li’s D* and F* summary statistics), values were mostly positive for the candidate vaccine antigens, except for *glurp* which had negative Tajima’s D values (Table S2 & Fig. S2), suggesting low frequency polymorphisms relative to that expected under neutrality. Tajima’s D values ranged from 4.113 for *surfin8.2* in Ghana to 0.456 for *msp1* in Madagascar; Fu&Li F* ranged from 4.551 for *surfin8.2* in Ghana to 1.069 for *phistb* in Kenya; and Fu&Li D* ranged from 3.521 for *surfin8.*2 in Ghana to 1.004 for *phistb* in Ethiopia (Table S2). The positive neutrality test values obtained for the antigens in this study is suggestive of a pattern of balancing selection for the candidate vaccine antigens across malaria endemic populations in sSA (Table 2). Comparison of Tajima’s D values for the candidate vaccine antigens analysed by subregions revealed that for most of the antigens there were no statistically significant differences (*P* > 0.05) between East versus West and Central Africa. This is further evidence of standing variation in these antigens across malaria endemic communities in sSA. As expected, there was strong correlation between Tajima’s D and Fu & Li’s F* values for all the antigens across the populations studied (r = 0.882). However, correlation was much lower between Tajima’s D and Fu & Li’s D* (r = 0.03), with some antigens, notably *glurp, celtos and phistb,* showing a negative correlation, while Fu & Li’s D* and F* had a weak correlation (r = 0.2) for the antigens except for *glurp* where Tajima’s D was negative but Fu and Li indices were positive (Fig. 2). Tajima’s D becomes negative when there is an excess of rare alleles resulting in more pairwise diversity than the number of segregating sites.

**Table 2:** Neutrality tests for candidate vaccine antigens.

Vaccine candidates	Tajima_D	Fu&Li_F*	Fu&Li_D*
**Pre-erythrocytic**			
celtos	0.45	1.59	1.9195
csp	0.599	1.4595	1.595
lsa3	0.872	1.419	1.3525
trap	1.658	2.3895	2.3015
**Erythrocytic**			
ama1	2.443	2.841	2.281
eba175	2.09	2.4335	1.9495
glurp	− 0.184	1.1895	1.7725
msp1	0.1915	1.8665	2.5675
phistb	0.6515	1.2785	1.2975
surfin8.2	2.9305	3.5335	2.9885
surfin14.1	0.76175	1.72825	1.9345

### Haplotype frequency and cluster network

**Figure 3:**
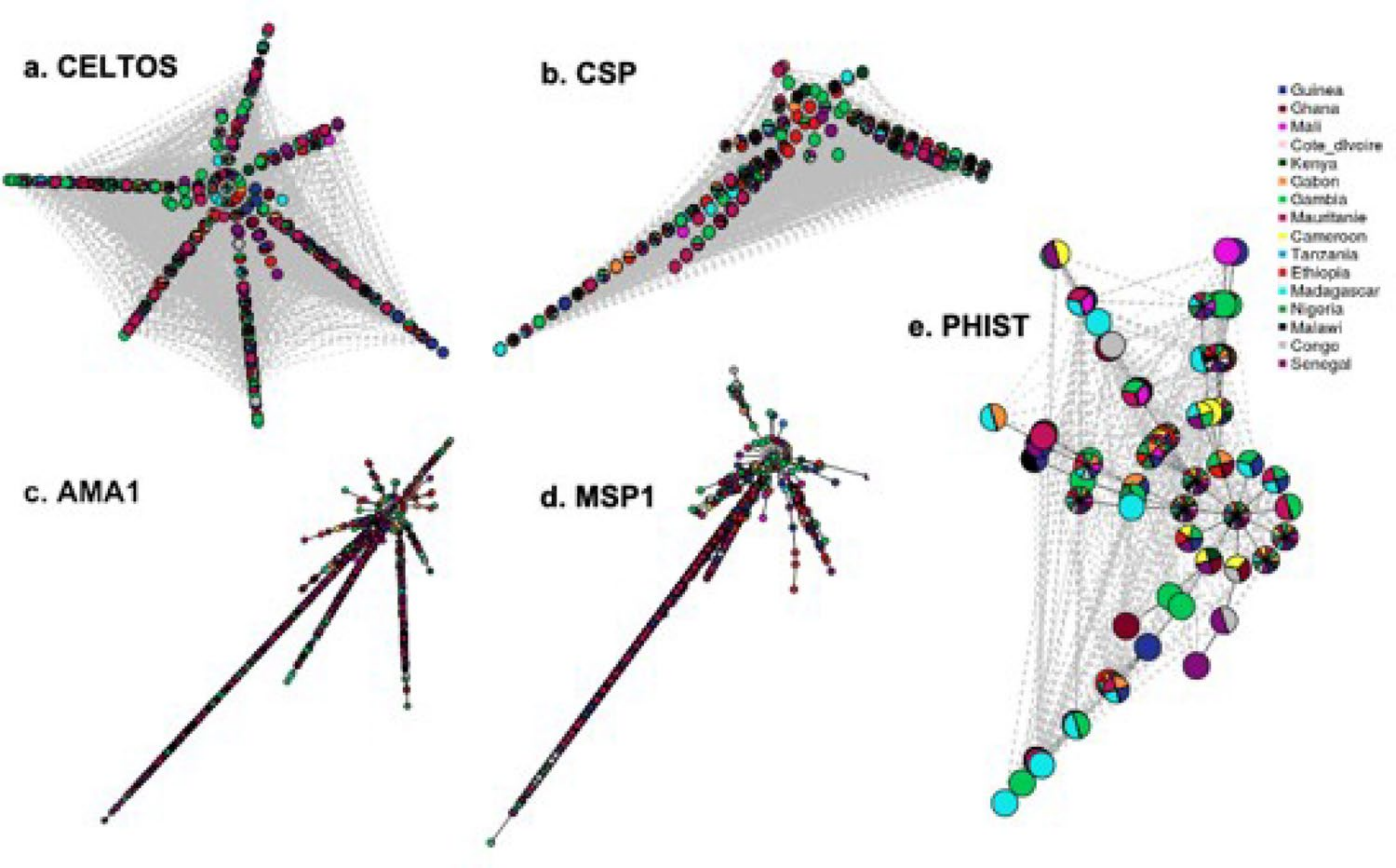
Representative haplotype cluster networks of pre-erythrocytic and erythrocytic vaccine candidates. Clustering of haplotypes across populations for pre-erythrocytic candidates (**a**) CELTOS, (**b**) CSP, and erythrocytic vaccine candidates (**c**) AMA1, (**d**) MSP1 and (**e**) PHISTB. Each node represents a haplotype with pies colour coded according to the proportion of haplotypes originating from a specific country. The lengths of edges connecting each pair of pies is proportion to the number of base pair substitutions separating the haplotypes.

The frequency of haplotypes for all antigens and details of haplotypes of each antigen per country are given in Table S3. To further understand the relatedness of the haplotypes across our populations, haplotype networks were constructed on selected vaccine candidates already under investigation in clinical trials; 2 pre-erythrocytic (*celtos* and *csp*) and 3 erythrocytic (*ama1*, *msp1* and *phistb*) antigens. The pre-erythrocytic antigens had a high number of haplotypes with no geographic clusters, suggesting lack of structure. Haplotype network of the erythrocytic antigens followed a similar pattern to the pre-erythrocytic antigens, except for *phistb* where geographic clustering of some haplotypes was evident (Fig. 3).

### Linkage disequilibrium

**Figure 4:**
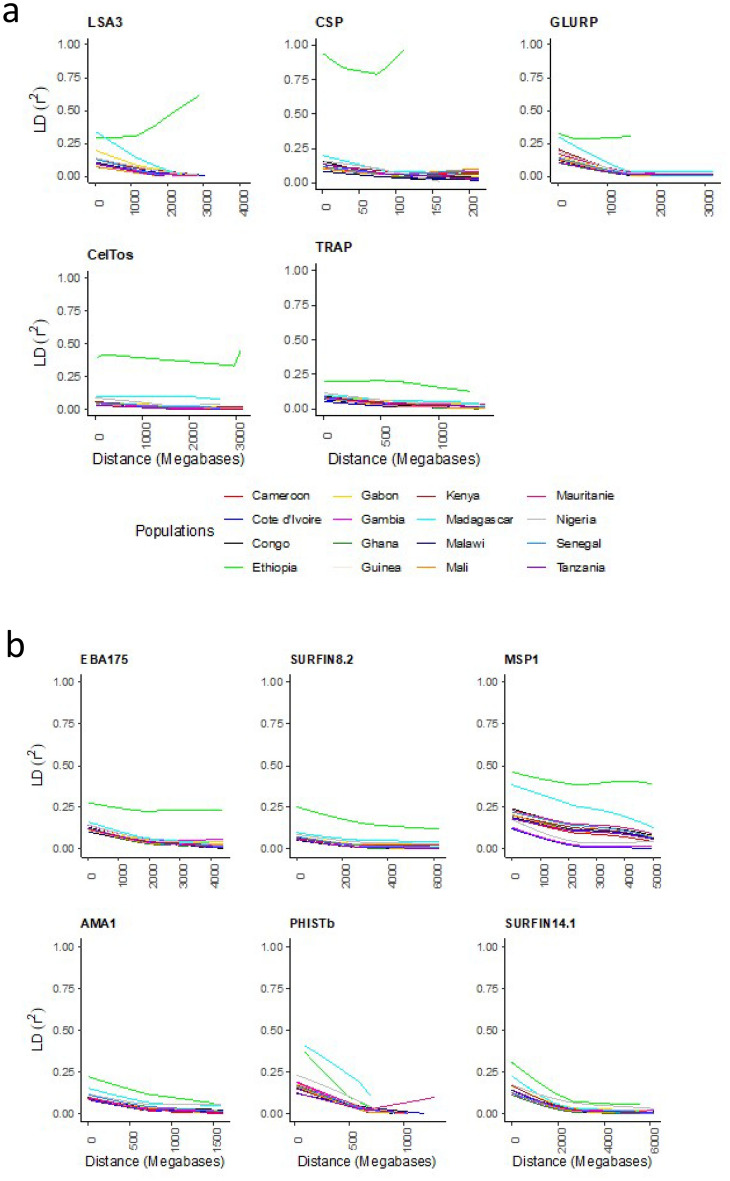
Linkage disequilibrium, (**a**) pre-erythrocytic and (**b**) erythrocytic vaccine candidates. R^2^ values decrease as distance increases. LD in Ethiopian samples for both (**a**) and (**b**) have a unique decay pattern.

Linkage Disequilibrium (LD) declined rapidly with physical separating pairs of SNPs for all antigens, falling below an r^2^ of 0.05 within 1000 base pairs (Fig. 4). Exceptionally, LD values were higher for Ethiopia, with r^2^ > 0.1 across most pairs of SNPs not significantly decaying with physical distance for most antigens. This observation may be underscored by variance in malaria transmission dynamics, population migration, demography and recombination potential within the SNPs in the genes analysed.

### Antigenic potential and 3D structure of phistb

**Figure 5:**
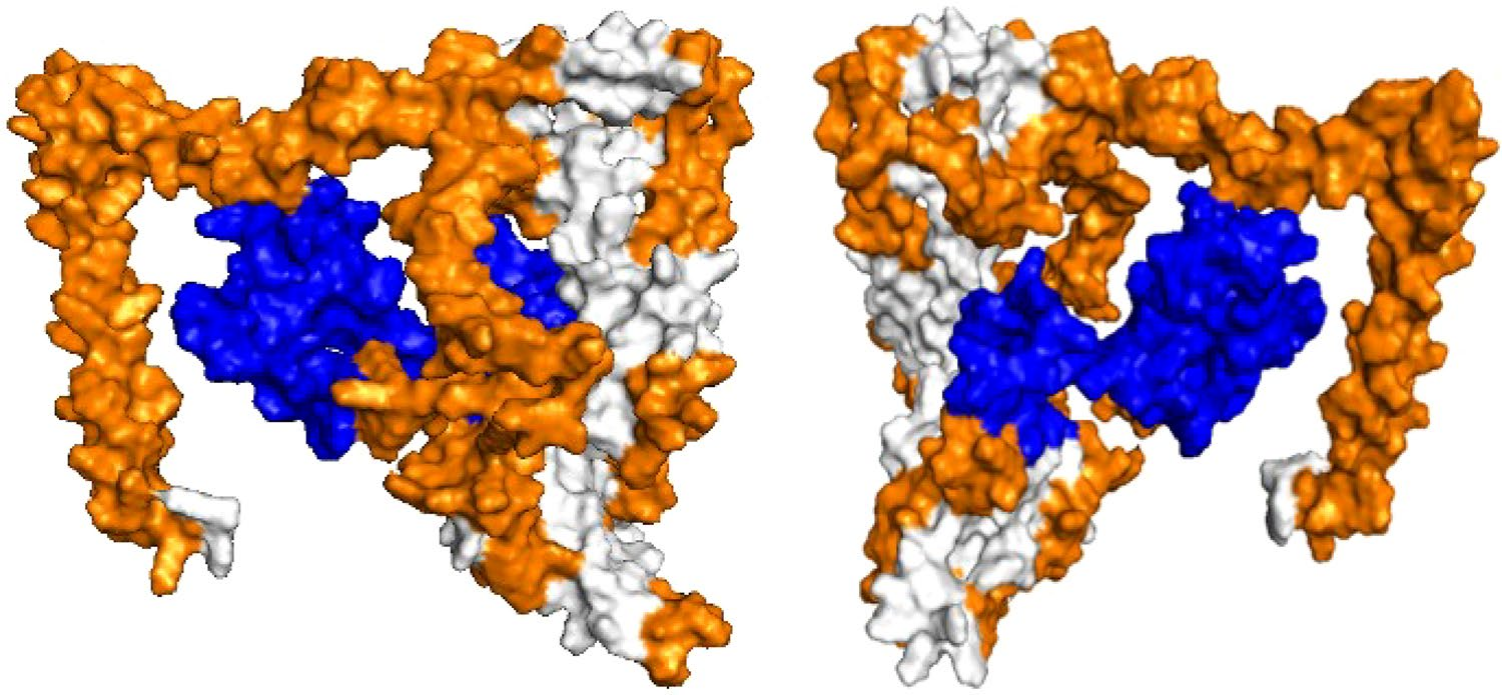
Left and right-handed three-dimensional structure of *phistb* from I-Tasser, mapping regions with positive Tajima’s D (blue) and B-cell epitopes in orange.

Epitope scanning for linear B cell epitopes on the antigens revealed several epitopes. Some of the regions with immune epitopes overlap with regions of high Tajima’s D, indicative of immune selection. The list of the B cell epitopes for the vaccine candidates and regions mapping with high Tajima’s D are provided in Table S4. We further explored the immunogenicity of *phistb,* which had only 2 predominant haplotypes (Table S3), and evidence of genetic structure between geographical parasite populations. The TMHMM server predicted one transmembrane helix between amino acid positions 54 and 73, and the likelihood of a signal peptide (Fig. S3). *Phistb* also has a post-translational modification- glycophosphatidyl inositol (GPI) site in its C-terminal region. GPI is used by proteins for anchoring to the plasma membrane (Fig. S4). Following 3D structure modelling of *Phistb* the linear B-cell epitopes predicted overlapped with regions with high positive Tajima’s D values (Fig. 5).

## Discussion

We carried out a large-scale population genetic analyses of candidate vaccine antigens and hitherto uncharacterised potential antigens using SNP data of *P. falciparum* isolates (n = 2375) from 16 African countries. Most advanced malaria vaccine candidates were initially designed without considering genetic diversity, resulting in poor protective efficacy as shown by FMP1/AS02A MSP1 or the AMA1 candidates that had limited vaccine allele representation in naturally circulating parasites, especially in Africa^11, 35, 36^. Therefore this focused analysis on African parasite populations is relevant, given intense but heterogenous transmission, and high parasite genetic diversity across sub-populations^3, 4^.

By screening for antigens with signatures of directional selection, we expected to identify those with dominant haplotypes and exposed immune epitopes, that could be included into the panel of candidates currently under development. We observed significant diversity amongst characterised vaccine candidates and new putative antigens across all populations, and this may result in poor efficacy both within clinical trials and in future deployment^37^. As immunity could be driving balancing selection signatures against specific domains of the antigens, our scan for signatures of selection localised these segments, with sequences of known immune epitopes that could be targeted for functional evaluation. However, antigens under high immune selection pressures and balancing selection are often highly polymorphic with moderate minor allele frequencies, likely to result in variance in allele specific immune response between populations^38^. This applies also to well-known erythrocytic vaccine candidates based on *ama1*, *msp1* and *eba175*, in which variation and selection has been a major hurdle in achieving broad efficacy across malaria endemic populations^35, 37, 39^. Like *msp1* and *ama1*, other erythrocytic antigens such as *surfin 8.2* and *surfin 14.1* showed signatures of both directional and balancing selection, the latter, also due to an excess of intermediate frequency variants that may result in vaccine escape. The *surfin* family of proteins are expressed by genes close to the sub-telomeres where other surface variant antigens (*pfemp1, rifin, stevor*) are encoded^5^. These are expressed on the surface of infected erythrocytes and are known targets of protective immunity and generating heterologous antibodies associated with reduction in febrile illness due to malaria infection^6^. *Surfin8.2* had the highest number of segregating sites and has been shown to be preferentially expressed in gametocytes^40, 41^. Being a PEXEL (*Plasmodium* export element) negatively exported protein with a transmembrane domain^42^, it will be exposed to the surface and can therefore be a target for transmission blocking vaccine development. Another transmission blocking vaccine*, glurp*, is in advanced development alone or as a multivalent component with other erythrocytic candidates such as *msp3* (GMZ2)^43^. Its lower Tajima’s D value and smaller number of haplotypes could provide an advantage for combination with other candidates such as *surfin8.2* to provide a broad range of erythrocytic protection and transmission blocking activity.

While erythrocytic antigens dominate the panel of malaria vaccine candidates in development, the only successful vaccine to date is the CSP-based RTS,S/AS-01. As shown already, the CSP gene is highly diverse, one of the factors contributing to reduced efficacy across populations. We assessed diversity at four other pre-erythrocytic candidates, *celtos, lsa3, pfsea* and *trap*. The least polymorphic one was the schizont egress antigen *pfsea,* whose antibodies have been associated with protection against high parasitemia and severe disease^44, 45^. It is under preclinical exploration in combination with invariant carboxyl of PfGARP and PfMSP1 in a tri-valent vaccine formulation. As previously described, *lsa3* also has a low number of haplotypes and is expressed on both sporozoites and the erythrocytic stages^46–48^. Thus it could also be considered as a blood stage vaccine candidate given its antibodies inhibit parasite growth in the erythrocyte^48^. Its further development against both erythrocytic and pre-erythrocytic stages will have to consider the African haplotypes described here. This also applies to one of the recent prominent candidates, *rh5*, which, despite a low number of haplotypes, had 12 non-synonymous SNPs and high between population differentiation determined by the fixation index *F*_ST_. Strong differentiation between eastern versus western African parasite populations would imply that population specific variation in haplotypes need to be considered for *rh5* vaccine development. Our findings also support previous studies which demonstrated that the number of haplotypes were consistently higher in countries with higher malaria transmission such as Cameroon, Mali and Malawi^49–51^. Therefore, continuous genetic screening for low diversity candidates to add to the pool of current antigens that can be combined in a multivalent malaria vaccine remains important.

We demonstrated the power of this genetic analysis by exemplifying one of the low diversity candidate antigens, *phistb*. PHIST proteins belong to the *Plasmodium* helical interspersed subtelomeric (PHIST) family made up of 65 gene members (PlasmoDB database, www.plasmodb.org) and are unified by possessing a single domain termed PHIST that is predicted to be composed solely of alpha helices^52, 53^. This family of exported proteins are conserved across the human *Plasmodium* species; *P. falciparum, P. vivax* and *P. knowlesi*^52^. As we identified a transmembrane domain and GPI anchors, we expect *phistb* to be surface anchored^54^. Previous studies have described *phistb* to localise to the host erythrocyte periphery through a PRESAN domain and an N-terminal sequence, and therefore exposed to immune interaction. Unsurprisingly, segments of the antigen had high Tajima’s D values, indicating balancing selection probably due to immune selection. These segments mapped to predicted B-cell epitopes which could be included in future candidate vaccine designs, preclinical testing and for selection of an optimal vaccine cocktail. The low genetic diversity, limited haplotypes in the population and linear B-cell epitopes of *phistb* are desirable features for designing an antimalarial vaccine that is less likely to produce allele-specific immune responses^2, 50^. Recent studies with in vitro expressed *phistb* protein demonstrated the presence of significant specific anti-*phistb* antibodies in children, with malaria specific immune responses^55^. This further supports predictions from genetic analysis and epitope mapping in line with other studies that have identified potential immune targets by modelling protein structures and predicting the functional relevance and implications of different domains as vaccine candidates^56, 57^. PHIST proteins are involved in remodelling infected red blood cells^54^. However, data on host antimalarial immune responses to these proteins, which could be crucial for vaccine development, is scarce.

Despite the relevant finding on top candidates like *rh5* and new potential candidates like *phistb*, our study has several limitations. Samples were collected mostly in one field site per country, and thus they may not provide an accurate representation of the circulating haplotypes across each country. In addition, the number of samples available from some countries such as Madagascar, Ethiopia and Nigeria, were small. Moreover, protocols for sample collection differed by country, including the time of sampling, which may influence the parasite haplotypes due to seasonality. The analysis was limited to high quality SNPs, which could have missed important genetic variations; and there is also the possibility of ascertainment bias in the dataset that have been utilised in this study, and more broader sampling for suspected vaccine candidate genes would be useful. However, despite these limitations, the analysis was comprehensive since we used a large dataset and provide a framework and basis for future studies and considerations. To verify our findings, functional studies and expanded genetic analysis incorporating targeted sequencing data are needed to further support our findings.

In conclusion, despite the broad genetic diversity of antigens of African *P. falciparum* isolates, it may be possible to identify the most prevalent antigen haplotypes within the continent for inclusion in a multivalent vaccine. Our findings demonstrate population genetic analysis can be used to identify antigens that consider African parasite genetic diversity for design of new vaccines.

## Supplementary Information

Supplementary Information.

## References

[1] Matuschewski K (2017). Vaccines against malaria-still a long way to go. FEBS J..

[2] Neafsey DE (2015). Genetic diversity and protective efficacy of the RTS, S/AS01 malaria vaccine. N. Engl. J. Med..

[3] Thera MA (2011). A field trial to assess a blood-stage malaria vaccine. N. Engl. J. Med..

[4] Pringle JC (2019). High plasmodium falciparum genetic diversity and temporal stability despite control efforts in high transmission settings along the international border between Zambia and the Democratic Republic of the Congo. Malar. J..

[5] Flück C (2004). Strain-specific humoral response to a polymorphic malaria vaccine. Infect. Immun..

[6] Genton B (2002). A recombinant blood-stage malaria vaccine reduces *Plasmodium falciparum* density and exerts selective pressure on parasite populations in a phase 1–2b trial in Papua New Guinea. J. Infect. Dis..

[7] Rastogi D (2007). Antigen-specific immune responses to influenza vaccine in utero. J. Clin. Invest..

[8] Ouattara A (2015). Designing malaria vaccines to circumvent antigen variability. Vaccine.

[9] Pringle JC (2018). RTS, S/AS01 malaria vaccine mismatch observed among Plasmodium falciparum isolates from southern and central Africa and globally. Sci. Rep..

[10] Salamanca DR (2019). Plasmodium falciparum blood stage antimalarial vaccines: An analysis of ongoing clinical trials and new perspectives related to synthetic vaccines. Front. Microbiol..

[11] Draper SJ (2018). Malaria vaccines: Recent advances and new horizons. Cell Host Microbe.

[12] Moser KA (2020). Strains used in whole organism Plasmodium falciparum vaccine trials differ in genome structure, sequence, and immunogenic potential. Genome Med..

[13] RTS, S Clinical Trials Partnership. Efficacy and safety of RTS,S/AS01 malaria vaccine with or without a booster dose in infants and children in Africa: final results of a phase 3, individually randomised, controlled trial. *Lancet Lond. Engl.* **386**, 31–45 (2015). 10.1016/S0140-6736(15)60721-8 PMC5626001 25913272

[14] Barry, A. E. & Arnott, A. Strategies for designing and monitoring malaria vaccines targeting diverse antigens. *Front. Immunol.* **5** (2014). 10.3389/fimmu.2014.00359 PMC4112938 25120545

[15] World Health Organization. Malaria vaccine rainbow tables (2013).

[16] Head MG (2017). Global funding trends for malaria research in sub-Saharan Africa: A systematic analysis. Lancet Glob. Health.

[17] Crompton PD, Pierce SK, Miller LH (2010). Advances and challenges in malaria vaccine development. J. Clin. Invest..

[18] Arama C, Troye-Blomberg M (2014). The path of malaria vaccine development: Challenges and perspectives. J. Intern. Med..

[19] Beeson JG (2019). Challenges and strategies for developing efficacious and long-lasting malaria vaccines. Sci. Transl. Med..

[20] Barry AE, Schultz L, Buckee CO, Reeder JC (2009). Contrasting population structures of the genes encoding ten leading vaccine-candidate antigens of the human malaria parasite, Plasmodium falciparum. PLoS ONE.

[21] Cortés A (2003). Geographical structure of diversity and differences between symptomatic and asymptomatic infections for Plasmodium falciparum vaccine candidate AMA1. Infect. Immun..

[22] Amambua-Ngwa A (2012). Population genomic scan for candidate signatures of balancing selection to guide antigen characterization in malaria parasites. PLoS Genet..

[23] Amambua-Ngwa A (2019). Major subpopulations of *Plasmodium falciparum* in sub-Saharan Africa. Science.

[24] Kamau E (2014). K13-propeller polymorphisms in plasmodium falciparum parasites from Sub-Saharan Africa. J. Infect. Dis..

[25] Pearson RD, Amato R, Kwiatkowski DP (2019). & MalariaGEN plasmodium falciparum community project. An open dataset of plasmodium falciparum genome variation in 7000 worldwide samples. Welcome Open Res..

[26] Pfeifer B, Wittelsbürger U, Ramos-Onsins SE, Lercher MJ (2014). PopGenome: An efficient Swiss Army Knife for population genomic analyses in R. Mol. Biol. Evol..

[27] Danecek P (2011). The variant call format and VCFtools. Bioinformatics.

[28] Chang CC (2015). Second-generation PLINK: Rising to the challenge of larger and richer datasets. GigaScience.

[29] Weir BS, Cockerham CC (1984). Estimating F-statistics for the analysis of population structure. Evolution.

[30] Goudet J (2005). hierfstat, a package for r to compute and test hierarchical F-statistics. Mol. Ecol. Notes.

[31] Paradis E (2010). Pegas: An R package for population genetics with an integrated-modular approach. Bioinformatics.

[32] Jespersen MC, Peters B, Nielsen M, Marcatili P (2017). BepiPred-2.0: Improving sequence-based B-cell epitope prediction using conformational epitopes. Nucleic Acids Res..

[33] Zhang Y (2008). I-TASSER server for protein 3D structure prediction. BMC Bioinform.

[34] Sonnhammer EL, von Heijne G, Krogh A (1998). A hidden Markov model for predicting transmembrane helices in protein sequences. Proc. Int. Conf. Intell. Syst. Mol. Biol..

[35] Escalante AA (2001). Polymorphism in the gene encoding the apical membrane antigen-1 (AMA-1) of Plasmodium falciparum. X. Asembo Bay Cohort Project. Mol. Biochem. Parasitol..

[36] Duan J (2008). Population structure of the genes encoding the polymorphic Plasmodium falciparum apical membrane antigen 1: Implications for vaccine design. Proc. Natl. Acad. Sci..

[37] Verra F (2006). Contrasting signatures of selection on the Plasmodium falciparum erythrocyte binding antigen gene family. Mol. Biochem. Parasitol..

[38] Conway DJ (2000). A principal target of human immunity to malaria identified by molecular population genetic and immunological analyses. Nat. Med..

[39] Beeson JG (2016). Merozoite surface proteins in red blood cell invasion, immunity and vaccines against malaria. FEMS Microbiol. Rev..

[40] Kanoi BN (2020). Global repertoire of human antibodies against plasmodium falciparum RIFINs, SURFINs, and STEVORs in a malaria exposed population. Front. Immunol..

[41] Winter G (2005). SURFIN is a polymorphic antigen expressed on Plasmodium falciparum merozoites and infected erythrocytes. J. Exp. Med..

[42] Morita M (2017). Immunoscreening of Plasmodium falciparum proteins expressed in a wheat germ cell-free system reveals a novel malaria vaccine candidate. Sci. Rep..

[43] Theisen M, Adu B, Mordmüller B, Singh S (2017). The GMZ2 malaria vaccine: From concept to efficacy in humans. Expert Rev. Vaccines.

[44] Raj DK (2014). Antibodies to PfSEA-1 block parasite egress from RBCs and protect against malaria infection. Science.

[45] Kurtis JD (2019). Maternally-derived antibodies to Schizont Egress antigen-1 and protection of infants from severe malaria. Clin. Infect. Dis..

[46] Daubersies P (2000). Protection against Plasmodium falciparum malaria in chimpanzees by immunization with the conserved pre-erythrocytic liver-stage antigen 3. Nat. Med..

[47] Longley RJ (2015). Comparative assessment of vaccine vectors encoding ten malaria antigens identifies two protective liver-stage candidates. Sci. Rep..

[48] Prieur E, Druilhe P (2009). The malaria candidate vaccine liver stage antigen-3 is highly conserved in Plasmodium falciparum isolates from diverse geographical areas. Malar. J..

[49] Anong DN, Nkuo-Akenji T, Fru-Cho J, Amambua-Ngwa A, Titanji VPK (2010). Genetic diversity of *Plasmodium falciparum* in Bolifamba, on the slopes of Mount Cameroon: Influence of MSP1 allelic variants on symptomatic malaria and anaemia. Ann. Trop. Med. Parasitol..

[50] Ouattara A (2018). Extent and dynamics of polymorphism in the malaria vaccine candidate plasmodium falciparum reticulocyte-binding protein homologue-5 in Kalifabougou, Mali. Am. J. Trop. Med. Hyg..

[51] Ocholla H (2014). Whole-genome scans provide evidence of adaptive evolution in malawian plasmodium falciparum isolates. J. Infect. Dis..

[52] Tarr SJ, Moon RW, Hardege I, Osborne AR (2014). A conserved domain targets exported PHISTb family proteins to the periphery of Plasmodium infected erythrocytes. Mol. Biochem. Parasitol..

[53] Sargeant TJ (2006). Lineage-specific expansion of proteins exported to erythrocytes in malaria parasites. Genome Biol..

[54] Oberli A (2014). A Plasmodium falciparum PHIST protein binds the virulence factor PfEMP1 and comigrates to knobs on the host cell surface. FASEB J. Off. Publ. Fed. Am. Soc. Exp. Biol..

[55] Isebe TI (2020). Molecular characterization of Plasmodium falciparum PHISTb proteins as potential targets of naturally-acquired immunity against malaria. Wellcome Open Res..

[56] Dutta S, Lee SY, Batchelor AH, Lanar DE (2007). Structural basis of antigenic escape of a malaria vaccine candidate. Proc. Natl. Acad. Sci..

[57] Takala SL (2009). Extreme polymorphism in a vaccine antigen and risk of clinical malaria: Implications for vaccine development. Sci. Transl. Med..

