# Supplementary material for: In silico characterisation of putative Plasmodium falciparum vaccine candidates in African malaria populations
Source: Sci Rep. 2021 Aug 10;11:16215. doi: 10.1038/s41598-021-95442-4 (PMC8355234; doi:10.1038/s41598-021-95442-4)
Supplement: Supplementary file 1 — Supplementary Information. [file 41598_2021_95442_MOESM1_ESM.docx]

**Title:** In silico characterisation of putative *Plasmodium falciparum* vaccine candidates in African malaria populations

Ajibola, O^1,2#^., Diop, M. F^1#^., Ghansah, A^3^., Amenga-Etego, L^4^., Golassa, L^5^., Apinjoh, T^6^., Randrianarivelojosia, M^7^., Maiga-Ascofare, O^8^., Yavo, W^9^., Bouyou-Akotet, M^10^., Oyebola, K. M^11^., Andagalu, B^12^., D’Alessandro, U^1^., Ishengoma, D^13^., Djimde, A. A^14^., Kamau, E*^15,16^., Amambua-Ngwa A^1^*.

^1^Medical Research Council Unit The Gambia at London School of Hygiene and Tropical Medicine, The Gambia

^2^First Technical University, Ibadan, Nigeria

^3^Noguchi Memorial Institute for Medical Research, University of Ghana, P.O. Box LG 581, Legon, Ghana

^4^West African Center for Cell Biology of Infectious Pathogens, University of Ghana, Accra, Ghana.

^5^Aklilu Lemma Institute of Pathobiology, Addis Ababa University, Addis Ababa, Ethiopia

^6^Department of Biochemistry and Molecular Biology, University of Buea, Buea, Cameroon

**^7^**Institut Pasteur of Madagascar, Antanarivo, Madagascar

^8^Bernhard Nocht Institute for Topical Medicine (BNITM), Hamburg, Germany

^9^Unite des Sciences Pharmaceutiques et Biologiques, University Félix Houphouët-Boigny, Abidjan, Côte d'Ivoire

^10^Faculty of Medicine, University of Health Sciences, Libreville, Gabon

^11^Department of Zoology, University of Lagos, Lagos, Nigeria

^12^United States Army Medical Research Directorate-Africa, Kenya Medical Research Institute/Walter Reed Project, Kisumu, Kenya

^13^National Institute for Medical Research (NIMR), Tanga, Tanzania

^14^Malaria Research and Training Centre, University of Science, Techniques and Technologies of Bamako, Bamako, Mali

^15^Department of Pathology and Laboratory Medicine, David Geffen School of Medicine, University of California, Los Angeles, California 90095, United States.

^16^U.S. Military HIV Research Program, Walter Reed Army Institute of Research, Silver Spring, MD, 20910, United States of America.

**Supplementary Table 1.** Haplotype diversity of *P. falciparum* vaccine candidates

| **Country** | **Pre-erythrocytic antigens** | **h** | **S** | **Pi** | **Hd** | **Theta** |
| --- | --- | --- | --- | --- | --- | --- |
| Cameroon | **Celtos** | 157 | 30 | 1.873800E-03 | 0.994 | 0.0018738 |
| Congo |  | 72 | 26 | 1.392900E-03 | 0.964 | 0.0013929 |
| Cote_dIvoire | | 53 | 27 | 1.686500E-03 | 0.985 | 0.0016865 |
| Ethiopia |  | 5 | 4 | 4.790000E-04 | 0.767 | 0.000479 |
| Gabon |  | 47 | 25 | 1.695000E-03 | 0.987 | 0.001695 |
| Gambia |  | 93 | 26 | 1.518400E-03 | 0.983 | 0.0015184 |
| Ghana |  | 155 | 30 | 1.770400E-03 | 0.991 | 0.0017704 |
| Guinea |  | 117 | 29 | 1.741200E-03 | 0.99 | 0.0017412 |
| Kenya |  | 55 | 23 | 1.662600E-03 | 0.995 | 0.0016626 |
| Madagascar |  | 18 | 21 | 1.467500E-03 | 0.975 | 0.0014675 |
| Malawi |  | 157 | 27 | 1.562900E-03 | 0.992 | 0.0015629 |
| Mali |  | 153 | 30 | 1.640900E-03 | 0.99 | 0.0016409 |
| Mauritania |  | 59 | 27 | 1.753300E-03 | 0.984 | 0.0017533 |
| Nigeria |  | 22 | 23 | 1.538100E-03 | 0.931 | 0.0015381 |
| Senegal |  | 66 | 26 | 1.549200E-03 | 0.972 | 0.0015492 |
| Tanzania |  | 145 | 30 | 1.711600E-03 | 0.989 | 0.0017116 |
|  |  |  |  |  |  |  |
|  |  |  |  |  |  |  |
| Cameroon | **CSP** | 72 | 17 | 1.5811200E-02 | 0.95 | 0.0117908 |
| Congo |  | 46 | 15 | 1.5801700E-02 | 0.951 | 0.0109115 |
| Cote_dIvoire | | 33 | 14 | 1.5364400E-02 | 0.948 | 0.0118617 |
| Ethiopia |  | 3 | 7 | 8.4227000E-03 | 0.29 | 0.0073027 |
| Gabon |  | 28 | 17 | 1.7534200E-02 | 0.964 | 0.014914 |
| Gambia |  | 53 | 18 | 1.6667400E-02 | 0.958 | 0.0275203 |
| Ghana |  | 82 | 18 | 1.5622000E-02 | 0.947 | 0.0117401 |
| Guinea |  | 59 | 18 | 1.5269300E-02 | 0.946 | 0.0129793 |
| Kenya |  | 35 | 16 | 1.4419400E-02 | 0.952 | 0.0139045 |
| Madagascar |  | 16 | 13 | 1.5211800E-02 | 0.96 | 0.0136882 |
| Malawi |  | 84 | 17 | 1.5906900E-02 | 0.956 | 0.0117401 |
| Mali |  | 66 | 18 | 1.5453600E-02 | 0.944 | 0.0125241 |
| Mauritania |  | 31 | 18 | 1.5495200E-02 | 0.931 | 0.0148241 |
| Nigeria |  | 13 | 10 | 1.2948700E-02 | 0.912 | 0.0116373 |
| Senegal |  | 33 | 17 | 1.5632500E-02 | 0.926 | 0.0128366 |
| Tanzania |  | 66 | 16 | 1.5681500E-02 | 0.952 | 0.0117401 |
|  |  |  |  |  |  |  |
|  |  |  |  |  |  |  |
| Cameroon | **LSA3** | 43 | 10 | 6.1730000E-04 | 0.87 | 0.000434 |
| Congo |  | 32 | 11 | 6.2880000E-04 | 0.876 | 0.0004471 |
| Cote_dIvoire | | 31 | 11 | 6.4040000E-04 | 0.893 | 0.000486 |
| Ethiopia |  | 8 | 9 | 8.9270000E-04 | 0.86 | 0.0004896 |
| Gabon |  | 19 | 9 | 6.2600000E-04 | 0.872 | 0.0004117 |
| Gambia |  | 32 | 11 | 5.9280000E-04 | 0.881 | 0.0005056 |
| Ghana |  | 41 | 12 | 5.1150000E-04 | 0.852 | 0.0005041 |
| Guinea |  | 36 | 11 | 5.9510000E-04 | 0.883 | 0.0005176 |
| Kenya |  | 21 | 9 | 5.8500000E-04 | 0.871 | 0.0004078 |
| Madagascar |  | 10 | 8 | 5.2970000E-04 | 0.616 | 0.0004941 |
| Malawi |  | 39 | 10 | 4.8880000E-04 | 0.836 | 0.0003601 |
| Mali |  | 44 | 12 | 5.6220000E-04 | 0.86 | 0.0005079 |
| Mauritania |  | 24 | 12 | 6.5100000E-04 | 0.882 | 0.0005583 |
| Nigeria |  | 18 | 11 | 7.4010000E-04 | 0.877 | 0.0005562 |
| Senegal |  | 28 | 11 | 5.8520000E-04 | 0.868 | 0.0005119 |
| Tanzania |  | 39 | 12 | 5.6960000E-04 | 0.859 | 0.0005041 |
|  |  |  |  |  |  |  |
|  |  |  |  |  |  |  |
| Cameroon | **Pfsea** | 2 | 1 | 1.7200000E-05 | 0.066 | 0.0000889 |
| Congo |  | 3 | 2 | 2.5800000E-05 | 0.069 | 0.0000998 |
| Cote_dIvoire | | 2 | 1 | 8.5000000E-06 | 0.057 | 0.0000543 |
| Ethiopia |  | 3 | 2 | 8.8000000E-05 | 0.29 | 0.0001336 |
| Gabon |  | 3 | 2 | 3.0300000E-05 | 0.101 | 0.0001124 |
| Gambia |  | 4 | 3 | 4.0900000E-05 | 0.123 | 0.0000733 |
| Ghana |  | 4 | 3 | 2.1800000E-05 | 0.073 | 0.0001327 |
| Guinea |  | 3 | 2 | 3.9600000E-05 | 0.131 | 0.0000978 |
| Kenya |  | 3 | 2 | 2.8900000E-05 | 0.157 | 0.000167 |
| Madagascar |  | 3 | 2 | 7.1100000E-05 | 0.236 | 0.0001349 |
| Malawi |  | 4 | 3 | 1.9400000E-05 | 0.059 | 0.0001327 |
| Mali |  | 3 | 2 | 1.2900000E-05 | 0.076 | 0.0000891 |
| Mauritania |  | 2 | 1 | 7.5000000E-06 | 0.05 | 0.0000528 |
| Nigeria |  | 1 | nd | nd | nd | nd |
| Senegal |  | 3 | 2 | 2.9900000E-05 | 0.099 | 0.0000967 |
| Tanzania |  | 4 | 3 | 4.3100000E-05 | 0.122 | 0.0001327 |
|  |  |  |  |  |  |  |
|  |  |  |  |  |  |  |
| Cameroon | **trap** | 190 | 43 | 8.0972000E-03 | 0.997 | 0.0046416 |
| Congo |  | 105 | 47 | 7.5930000E-03 | 0.999 | 0.0056901 |
| Cote_dIvoire | | 62 | 42 | 8.8154000E-03 | 0.995 | 0.0054124 |
| Ethiopia |  | 9 | 22 | 4.3955000E-03 | 0.82 | 0.0034908 |
| Gabon |  | 48 | 40 | 7.9896000E-03 | 0.992 | 0.0054708 |
| Gambia |  | 119 | 45 | 8.2968000E-03 | 0.99 | 0.0052713 |
| Ghana |  | 199 | 46 | 8.8850000E-03 | 0.999 | 0.0050418 |
| Guinea |  | 144 | 46 | 7.7104000E-03 | 0.998 | 0.0053417 |
| Kenya |  | 58 | 47 | 8.1132000E-03 | 0.998 | 0.005948 |
| Madagascar |  | 23 | 40 | 7.9844000E-03 | 0.996 | 0.0060856 |
| Malawi |  | 209 | 48 | 8.3857000E-03 | 0.998 | 0.0049367 |
| Mali |  | 211 | 46 | 8.5751000E-03 | 0.998 | 0.004868 |
| Mauritania |  | 66 | 44 | 8.2805000E-03 | 0.993 | 0.0055115 |
| Nigeria |  | 30 | 43 | 9.3090000E-03 | 0.998 | 0.00649 |
| Senegal |  | 86 | 46 | 8.5676000E-03 | 0.981 | 0.0053978 |
| Tanzania |  | 179 | 50 | 9.0535000E-03 | 0.996 | 0.0053569 |
|  |  |  |  |  |  |  |
|  | **Erythrocytic antigens** | | |  |  |  |
| Cameroon | **ama1** | 184 | 46 | 1.8738000E-03 | 0.996 | 0.004199 |
| Congo |  | 96 | 44 | 1.3929000E-03 | 0.995 | 0.0045134 |
| Cote_dIvoire | | 61 | 45 | 1.6865000E-03 | 0.995 | 0.0050179 |
| Ethiopia |  | 11 | 32 | 4.7900000E-04 | 0.88 | 0.0043937 |
| Gabon |  | 48 | 42 | 1.6950000E-03 | 0.993 | 0.0048494 |
| Gambia |  | 119 | 46 | 1.5184000E-03 | 0.996 | 0.0044773 |
| Ghana |  | 193 | 47 | 1.7704000E-03 | 0.998 | 0.00409 |
| Guinea |  | 137 | 46 | 1.7412000E-03 | 0.997 | 0.0044594 |
| Kenya |  | 58 | 42 | 1.6626000E-03 | 0.998 | 0.0048037 |
| Madagascar |  | 22 | 39 | 1.4675000E-03 | 0.993 | 0.0054046 |
| Malawi |  | 203 | 46 | 1.5629000E-03 | 0.997 | 0.0041809 |
| Mali |  | 195 | 47 | 1.6409000E-03 | 0.998 | 0.0043095 |
| Mauritania |  | 67 | 46 | 1.7533000E-03 | 0.994 | 0.005019 |
| Nigeria |  | 30 | 44 | 1.5381000E-03 | 0.998 | 0.0057621 |
| Senegal |  | 81 | 45 | 1.5492000E-03 | 0.985 | 0.004472 |
| Tanzania |  | 171 | 46 | 1.7116000E-03 | 0.992 | 0.0041809 |
|  |  |  |  |  |  |  |
|  |  |  |  |  |  |  |
| Cameroon | **chrom3** | 10 | 4 | 4.9635000E-03 | 0.669 | 0.0034121 |
| Congo |  | 8 | 4 | 5.2496000E-03 | 0.689 | 0.0038343 |
| Cote_dIvoire | | 4 | 3 | 4.5032000E-03 | 0.641 | 0.0031261 |
| Ethiopia |  | 1 |  | nd | nd | nd |
| Gabon |  | 4 | 3 | 5.3490000E-03 | 0.716 | 0.0032369 |
| Gambia |  | 7 | 4 | 4.8374000E-03 | 0.657 | 0.0036382 |
| Ghana |  | 6 | 4 | 3.9654000E-03 | 0.628 | 0.0033974 |
| Guinea |  | 8 | 4 | 4.4594000E-03 | 0.621 | 0.0036237 |
| Kenya |  | 7 | 5 | 4.3541000E-03 | 0.675 | 0.005344 |
| Madagascar |  | 6 | 5 | 5.7267000E-03 | 0.71 | 0.006475 |
| Malawi |  | 11 | 5 | 4.6523000E-03 | 0.609 | 0.0042467 |
| Mali |  | 8 | 4 | 5.1576000E-03 | 0.669 | 0.0034274 |
| Mauritania |  | 5 | 3 | 4.7041000E-03 | 0.679 | 0.0030588 |
| Nigeria |  | 4 | 3 | 5.4219000E-03 | 0.723 | 0.0036713 |
| Senegal |  | 7 | 4 | 4.8975000E-03 | 0.656 | 0.0037147 |
| Tanzania |  | 10 | 5 | 4.4716000E-03 | 0.624 | 0.0042467 |
|  |  |  |  |  |  |  |
|  |  |  |  |  |  |  |
| Cameroon | **eba175** | 173 | 29 | 1.8831000E-03 | 0.993 | 0.0010065 |
| Congo |  | 96 | 28 | 1.8210000E-03 | 0.994 | 0.0010556 |
| Cote_dIvoire | | 60 | 27 | 1.8894000E-03 | 0.995 | 0.0011476 |
| Ethiopia |  | 12 | 21 | 1.7138000E-03 | 0.9 | 0.0010597 |
| Gabon |  | 48 | 28 | 1.7804000E-03 | 0.993 | 0.0011882 |
| Gambia |  | 101 | 27 | 1.8125000E-03 | 0.979 | 0.0009238 |
| Ghana |  | 177 | 29 | 1.7181000E-03 | 0.995 | 0.0009688 |
| Guinea |  | 132 | 29 | 1.9137000E-03 | 0.995 | 0.001071 |
| Kenya |  | 56 | 29 | 1.7708000E-03 | 0.997 | 0.0012191 |
| Madagascar |  | 22 | 26 | 1.8243000E-03 | 0.993 | 0.0012733 |
| Malawi |  | 188 | 30 | 1.8665000E-03 | 0.996 | 0.0010022 |
| Mali |  | 176 | 29 | 1.8040000E-03 | 0.994 | 0.000976 |
| Mauritania |  | 64 | 28 | 1.8051000E-03 | 0.988 | 0.0011155 |
| Nigeria |  | 24 | 26 | 1.7127000E-03 | 0.976 | 0.0012197 |
| Senegal |  | 79 | 29 | 1.8836000E-03 | 0.98 | 0.0010592 |
| Tanzania |  | 169 | 30 | 1.9243000E-03 | 0.993 | 0.0010022 |
|  |  |  |  |  |  |  |
|  |  |  |  |  |  |  |
| Cameroon | **glurp** | 74 | 24 | 9.2680000E-04 | 0.95 | 0.0015919 |
| Congo |  | 50 | 20 | 9.9870000E-04 | 0.96 | 0.0013154 |
| Cote_dIvoire | | 39 | 22 | 1.1775000E-03 | 0.961 | 0.0017731 |
| Ethiopia |  | 6 | 5 | 5.4390000E-04 | 0.79 | 0.0007747 |
| Gabon |  | 30 | 19 | 9.9490000E-04 | 0.966 | 0.0015398 |
| Gambia |  | 56 | 23 | 9.6050000E-04 | 0.931 | 0.0014802 |
| Ghana |  | 71 | 24 | 9.6060000E-04 | 0.957 | 0.0015851 |
| Guinea |  | 61 | 21 | 1.0694000E-03 | 0.952 | 0.0015462 |
| Kenya |  | 36 | 18 | 9.8510000E-04 | 0.957 | 0.001408 |
| Madagascar |  | 14 | 16 | 1.0728000E-03 | 0.957 | 0.0014927 |
| Malawi |  | 81 | 21 | 9.7310000E-04 | 0.954 | 0.001352 |
| Mali |  | 85 | 24 | 9.6890000E-04 | 0.944 | 0.001597 |
| Mauritania |  | 34 | 21 | 1.0123000E-03 | 0.957 | 0.0015567 |
| Nigeria |  | 25 | 20 | 1.2345000E-03 | 0.972 | 0.0016367 |
| Senegal |  | 43 | 23 | 1.0183000E-03 | 0.941 | 0.0014783 |
| Tanzania |  | 70 | 23 | 1.0284000E-03 | 0.951 | 0.0014919 |
|  |  |  |  |  |  |  |
|  |  |  |  |  |  |  |
| Cameroon | **hyp9** | 4 | 3 | 2.3630000E-04 | 0.042 | 0.0007586 |
| Congo |  | 4 | 3 | 2.0640000E-04 | 0.052 | 0.0008524 |
| Cote_dIvoire | | 2 | 1 | 9.5200000E-05 | 0 | 0.0003089 |
| Ethiopia |  | 2 | 2 | 5.1180000E-04 | 0.153 | 0.0007607 |
| Gabon |  | 2 | 2 | 1.1650000E-04 | 0.034 | 0.0006397 |
| Gambia |  | 3 | 2 | 2.7230000E-04 | 0.074 | 0.0005392 |
| Ghana |  | 4 | 4 | 3.0380000E-04 | 0.032 | 0.0007553 |
| Guinea |  | 6 | 4 | 4.1200000E-04 | 0.086 | 0.0010741 |
| Kenya |  | 4 | 3 | 2.7140000E-04 | 0.064 | 0.0009505 |
| Madagascar |  | 2 | 3 | 4.1680000E-04 | 0.083 | 0.0011516 |
| Malawi |  | 5 | 4 | 2.3360000E-04 | 0.066 | 0.0010071 |
| Mali |  | 3 | 2 | 1.2940000E-04 | 0.017 | 0.000508 |
| Mauritania |  | 4 | 3 | 2.1210000E-04 | 0.05 | 0.0009067 |
| Nigeria |  | 3 | 2 | 4.1080000E-04 | 0.065 | 0.0007255 |
| Senegal |  | 5 | 4 | 3.6040000E-04 | 0.085 | 0.0011011 |
| Tanzania |  | 4 | 4 | 2.2900000E-04 | 0.021 | 0.0010071 |
|  |  |  |  |  |  |  |
|  |  |  |  |  |  |  |
| Cameroon | **hyp10** | 31 | 10 | 1.5897100E-02 | 0.833 | 0.0115059 |
| Congo |  | 25 | 8 | 1.5832600E-02 | 0.799 | 0.0103436 |
| Cote_dIvoire | | 18 | 9 | 1.6797700E-02 | 0.882 | 0.0126498 |
| Ethiopia |  | 4 | 5 | 1.6301200E-02 | 0.667 | 0.0086533 |
| Gabon |  | 13 | 8 | 1.6642800E-02 | 0.814 | 0.0116428 |
| Gambia |  | 24 | 10 | 1.8940400E-02 | 0.85 | 0.0122684 |
| Ghana |  | 41 | 10 | 1.8057000E-02 | 0.857 | 0.0114563 |
| Guinea |  | 27 | 9 | 1.6819800E-02 | 0.844 | 0.0109974 |
| Kenya |  | 20 | 9 | 1.3972600E-02 | 0.764 | 0.0129748 |
| Madagascar |  | 10 | 9 | 1.4239400E-02 | 0.754 | 0.0157206 |
| Malawi |  | 40 | 10 | 1.3693700E-02 | 0.777 | 0.0114563 |
| Mali |  | 40 | 10 | 1.7584300E-02 | 0.8 | 0.0115574 |
| Mauritania |  | 19 | 10 | 1.5425100E-02 | 0.791 | 0.0137528 |
| Nigeria |  | 9 | 9 | 1.5118500E-02 | 0.789 | 0.0148559 |
| Senegal |  | 20 | 9 | 1.8244300E-02 | 0.828 | 0.0112737 |
| Tanzania |  | 30 | 10 | 1.4925900E-02 | 0.743 | 0.0114563 |
|  |  |  |  |  |  |  |
|  |  |  |  |  |  |  |
| Cameroon | **msp1** | 225 | 84 | 3.2007000E-03 | 0.999 | 0.0025317 |
| Congo |  | 106 | 63 | 2.8782000E-03 | 0.998 | 0.0021171 |
| Cote_dIvoire | | 67 | 93 | 3.5311000E-03 | 0.999 | 0.0033803 |
| Ethiopia |  | 21 | 74 | 6.1019000E-03 | 0.973 | 0.0034537 |
| Gabon |  | 55 | 95 | 3.2226000E-03 | 0.998 | 0.0035374 |
| Gambia |  | 131 | 64 | 3.0152000E-03 | 0.996 | 0.0027919 |
| Ghana |  | 218 | 97 | 3.0258000E-03 | 1 | 0.0029018 |
| Guinea |  | 158 | 98 | 3.5068000E-03 | 1 | 0.0032081 |
| Kenya |  | 58 | 91 | 3.2019000E-03 | 0.997 | 0.0032827 |
| Madagascar |  | 21 | 72 | 2.8020000E-03 | 0.986 | 0.003173 |
| Malawi |  | 240 | 98 | 2.8902000E-03 | 1 | 0.0028725 |
| Mali |  | 227 | 98 | 3.1005000E-03 | 1 | 0.0028646 |
| Mauritania |  | 72 | 93 | 3.0298000E-03 | 0.996 | 0.0033906 |
| Nigeria |  | 31 | 58 | 3.0013000E-03 | 1 | 0.002552 |
| Senegal |  | 114 | 96 | 3.1892000E-03 | 0.994 | 0.0031087 |
| Tanzania |  | 217 | 99 | 2.9735000E-03 | 0.999 | 0.0029018 |
|  |  |  |  |  |  |  |
|  |  |  |  |  |  |  |
| Cameroon | **phistb** | 31 | 11 | 1.6512000E-03 | 0.864 | 0.0012388 |
| Congo |  | 23 | 10 | 1.4293000E-03 | 0.809 | 0.0012655 |
| Cote_dIvoire | | 15 | 9 | 1.5892000E-03 | 0.824 | 0.0012381 |
| Ethiopia |  | 4 | 4 | 9.9100000E-04 | 0.677 | 0.0006776 |
| Gabon |  | 15 | 11 | 1.5994000E-03 | 0.826 | 0.0015669 |
| Gambia |  | 27 | 10 | 1.6740000E-03 | 0.858 | 0.0012008 |
| Ghana |  | 35 | 11 | 1.6421000E-03 | 0.881 | 0.0012334 |
| Guinea |  | 26 | 11 | 1.6464000E-03 | 0.884 | 0.0013156 |
| Kenya |  | 12 | 8 | 1.2483000E-03 | 0.736 | 0.0011288 |
| Madagascar |  | 7 | 5 | 1.1838000E-03 | 0.783 | 0.0008548 |
| Malawi |  | 31 | 10 | 1.4076000E-03 | 0.794 | 0.0011213 |
| Mali |  | 27 | 11 | 1.6022000E-03 | 0.879 | 0.0012443 |
| Mauritania |  | 20 | 10 | 1.6771000E-03 | 0.878 | 0.0013461 |
| Nigeria |  | 12 | 7 | 1.7558000E-03 | 0.841 | 0.0011309 |
| Senegal |  | 16 | 10 | 1.7967000E-03 | 0.877 | 0.001226 |
| Tanzania |  | 25 | 10 | 1.4270000E-03 | 0.802 | 0.0011213 |
|  |  |  |  |  |  |  |
|  |  |  |  |  |  |  |
| Cameroon | **rh5** | 8 | 5 | 5.7450000E-04 | 0.404 | 0.0009502 |
| Congo |  | 10 | 5 | 9.0650000E-04 | 0.476 | 0.0010678 |
| Cote_dIvoire | | 7 | 5 | 7.2010000E-04 | 0.452 | 0.0011608 |
| Ethiopia |  | 5 | 4 | 1.3212000E-03 | 0.617 | 0.0011434 |
| Gabon |  | 6 | 5 | 5.5060000E-04 | 0.395 | 0.0012019 |
| Gambia |  | 5 | 3 | 7.5100000E-04 | 0.544 | 0.0004946 |
| Ghana |  | 9 | 5 | 7.6130000E-04 | 0.42 | 0.0009461 |
| Guinea |  | 10 | 5 | 9.1000000E-04 | 0.588 | 0.001046 |
| Kenya |  | 12 | 5 | 1.4586000E-03 | 0.721 | 0.0011906 |
| Madagascar |  | 3 | 2 | 1.3031000E-03 | 0.562 | 0.000577 |
| Malawi |  | 9 | 5 | 1.3073000E-03 | 0.668 | 0.0009461 |
| Mali |  | 10 | 5 | 8.4760000E-04 | 0.573 | 0.0009533 |
| Mauritania |  | 6 | 4 | 8.9360000E-04 | 0.585 | 0.000677 |
| Nigeria |  | 4 | 3 | 5.1460000E-04 | 0.385 | 0.0010629 |
| Senegal |  | 5 | 3 | 7.1650000E-04 | 0.509 | 0.0006207 |
| Tanzania |  | 11 | 5 | 1.3451000E-03 | 0.591 | 0.0009461 |
|  |  |  |  |  |  |  |
|  |  |  |  |  |  |  |
| Cameroon | **surfin8.2** | 224 | 158 | 8.2020000E-03 | 0.999 | 0.0037709 |
| Congo |  | 111 | 157 | 8.0489000E-03 | 1 | 0.0042107 |
| Cote_d’Ivoire | | 65 | 151 | 8.2289000E-03 | 0.998 | 0.0044024 |
| Ethiopia |  | 11 | 124 | 7.4085000E-03 | 0.9 | 0.0044514 |
| Gabon |  | 55 | 150 | 7.9778000E-03 | 0.998 | 0.0045282 |
| Gambia |  | 140 | 157 | 7.9593000E-03 | 0.999 | 0.0039954 |
| Ghana |  | 222 | 157 | 8.2748000E-03 | 1 | 0.0037071 |
| Guinea |  | 155 | 155 | 8.1548000E-03 | 0.999 | 0.0039287 |
| Kenya |  | 61 | 150 | 8.1249000E-03 | 1 | 0.0044856 |
| Madagascar |  | 23 | 146 | 8.1305000E-03 | 0.996 | 0.0052899 |
| Malawi |  | 240 | 160 | 8.0604000E-03 | 1 | 0.0038022 |
| Mali |  | 228 | 157 | 8.0761000E-03 | 1 | 0.0037638 |
| Mauritania |  | 76 | 156 | 8.1300000E-03 | 0.999 | 0.0044502 |
| Nigeria |  | 31 | 146 | 8.0101000E-03 | 1 | 0.004999 |
| Senegal |  | 109 | 156 | 8.0534000E-03 | 0.993 | 0.0040534 |
| Tanzania |  | 221 | 160 | 8.0973000E-03 | 0.999 | 0.0038022 |
|  |  |  |  |  |  |  |
|  |  |  |  |  |  |  |
| Cameroon | **surfin14.1** | 182 | 33 | 1.5252000E-03 | 0.993 | 0.0007944 |
| Congo |  | 98 | 34 | 1.5147000E-03 | 0.997 | 0.0009197 |
| Cote_dIvoire | | 63 | 32 | 1.6536000E-03 | 0.998 | 0.000941 |
| Ethiopia |  | 18 | 25 | 1.3430000E-03 | 0.95 | 0.0009052 |
| Gabon |  | 48 | 35 | 1.5695000E-03 | 0.988 | 0.0010657 |
| Gambia |  | 121 | 33 | 1.4318000E-03 | 0.994 | 0.000847 |
| Ghana |  | 195 | 36 | 1.5137000E-03 | 0.998 | 0.0008628 |
| Guinea |  | 136 | 32 | 1.5001000E-03 | 0.997 | 0.0008181 |
| Kenya |  | 59 | 32 | 1.6049000E-03 | 0.999 | 0.0009652 |
| Madagascar |  | 23 | 27 | 1.6902000E-03 | 0.996 | 0.0009867 |
| Malawi |  | 218 | 35 | 1.6142000E-03 | 0.998 | 0.0008389 |
| Mali |  | 185 | 32 | 1.4823000E-03 | 0.998 | 0.0007737 |
| Mauritania |  | 64 | 35 | 1.5488000E-03 | 0.995 | 0.001007 |
| Nigeria |  | 29 | 28 | 1.5602000E-03 | 0.994 | 0.0009669 |
| Senegal |  | 101 | 35 | 1.4896000E-03 | 0.992 | 0.0009172 |
| Tanzania |  | 208 | 35 | 1.7387000E-03 | 0.998 | 0.0008389 |
|  | nd, not determined; h, number of haplotypes; s, number of segregating sites; Hd, haplotype diversity | | |  |  |  |

**Supplementary Table 2**. Neutrality tests for vaccine candidates

| **Country** | **Pre-erythrocytic antigens** | **TajimaD** | **Fu&Li_F*** | **Fu&Li_D*** |
| --- | --- | --- | --- | --- |
| Cameroon | *celtos* | 0.87 | 1.841 | 2.014 |
| Congo |  | 0.044 | 1.382 | 1.917 |
| Cote_dIvoire | | 0.26 | 1.51 | 1.921 |
| Ethiopia |  | 1.581 | 1.379 | 1.004 |
| Gabon |  | 0.417 | 1.559 | 1.865 |
| Gambia |  | 0.446 | 1.573 | 1.917 |
| Ghana |  | 1.019 | 1.898 | 1.972 |
| Guinea |  | 0.553 | 1.67 | 1.994 |
| Kenya |  | 0.661 | 1.644 | 1.822 |
| Madagascar |  | -0.061 | 1.296 | 1.7 |
| Malawi |  | 0.664 | 1.684 | 1.929 |
| Mali |  | 0.423 | 1.607 | 2.015 |
| Mauritania |  | 0.451 | 1.602 | 1.929 |
| Nigeria |  | -0.021 | 1.336 | 1.765 |
| Senegal |  | 0.449 | 1.578 | 1.918 |
| Tanzania |  | 0.63 | 1.71 | 2.009 |
|  |  |  |  |  |
| Cameroon | *csp* | 0.817 | 1.561 | 1.599 |
| Congo |  | 0.883 | 1.565 | 1.562 |
| Cote_dIvoire | | 0.773 | 1.502 | 1.541 |
| Ethiopia |  | 0.406 | 1.145 | 1.242 |
| Gabon |  | 0.487 | 1.455 | 1.653 |
| Gambia |  | 0.634 | 1.514 | 1.663 |
| Ghana |  | 0.929 | 1.607 | 1.576 |
| Guinea |  | 0.381 | 1.391 | 1.662 |
| Kenya |  | 0.101 | 1.259 | 1.619 |
| Madagascar |  | 0.336 | 1.322 | 1.511 |
| Malawi |  | 0.895 | 1.593 | 1.591 |
| Mali |  | 0.564 | 1.464 | 1.641 |
| Mauritania |  | 0.104 | 1.292 | 1.685 |
| Nigeria |  | 0.83 | 1.422 | 1.392 |
| Senegal |  | 0.549 | 1.454 | 1.632 |
| Tanzania |  | 1.216 | 1.719 | 1.546 |
|  |  |  |  |  |
| Cameroon | *lsa3* | 1.503 | 1.637 | 1.266 |
| Congo |  | 0.957 | 1.464 | 1.376 |
| Cote_dIvoire | | 0.792 | 1.412 | 1.405 |
| Ethiopia |  | 2.305 | 1.957 | 1.352 |
| Gabon |  | 1.271 | 1.54 | 1.309 |
| Gambia |  | 0.903 | 1.426 | 1.353 |
| Ghana |  | 0.884 | 1.4 | 1.308 |
| Guinea |  | 0.925 | 1.435 | 1.351 |
| Kenya |  | 1.053 | 1.447 | 1.306 |
| Madagascar |  | 0.566 | 1.253 | 1.302 |
| Malawi |  | 0.86 | 1.342 | 1.257 |
| Mali |  | 0.646 | 1.328 | 1.376 |
| Mauritania |  | 0.638 | 1.375 | 1.448 |
| Nigeria |  | 0.833 | 1.457 | 1.437 |
| Senegal |  | 0.809 | 1.39 | 1.362 |
| Tanzania |  | 0.846 | 1.412 | 1.36 |
|  |  |  |  |  |
| Cameroon | *trap* | 2.151 | 2.699 | 2.298 |
| Congo |  | 1.062 | 2.115 | 2.305 |
| Cote_dIvoire | | 1.905 | 2.471 | 2.166 |
| Ethiopia |  | 0.836 | 1.678 | 1.722 |
| Gabon |  | 1.528 | 2.246 | 2.107 |
| Gambia |  | 1.815 | 2.518 | 2.311 |
| Ghana |  | 2.778 | 3.111 | 2.348 |
| Guinea |  | 1.409 | 2.308 | 2.329 |
| Kenya |  | 0.948 | 2.015 | 2.195 |
| Madagascar |  | 0.843 | 1.818 | 1.908 |
| Malawi |  | 1.851 | 2.588 | 2.39 |
| Mali |  | 2.099 | 2.702 | 2.351 |
| Mauritania |  | 1.481 | 2.286 | 2.211 |
| Nigeria |  | 1.431 | 2.131 | 1.996 |
| Senegal |  | 1.788 | 2.505 | 2.314 |
| Tanzania |  | 2.079 | 2.738 | 2.426 |
|  |  | 1.63 |  |  |
|  | **Erythrocytic antigens** |  |  |  |
| Cameroon | *ama1* | 2.816 | 3.102 | 2.352 |
| Congo |  | 2.459 | 2.827 | 2.263 |
| Cote_dIvoire | | 2.397 | 2.743 | 2.202 |
| Ethiopia |  | 1.196 | 1.925 | 1.852 |
| Gabon |  | 2.434 | 2.706 | 2.131 |
| Gambia |  | 2.418 | 2.855 | 2.326 |
| Ghana |  | 3.202 | 3.368 | 2.368 |
| Guinea |  | 2.452 | 2.875 | 2.329 |
| Kenya |  | 2.358 | 2.677 | 2.141 |
| Madagascar |  | 1.843 | 2.239 | 1.901 |
| Malawi |  | 2.911 | 3.162 | 2.355 |
| Mali |  | 2.675 | 3.033 | 2.368 |
| Mauritania |  | 2.12 | 2.628 | 2.236 |
| Nigeria |  | 1.862 | 2.33 | 2.003 |
| Senegal |  | 2.808 | 3.043 | 2.299 |
| Tanzania |  | 3.032 | 3.233 | 2.357 |
|  |  |  |  |  |
| Cameroon | *eba175* | 2.447 | 2.653 | 1.988 |
| Congo |  | 2.011 | 2.391 | 1.966 |
| Cote_dIvoire | | 2.049 | 2.367 | 1.921 |
| Ethiopia |  | 1.98 | 2.136 | 1.705 |
| Gabon |  | 1.48 | 2.098 | 1.925 |
| Gambia |  | 2.35 | 2.555 | 1.944 |
| Ghana |  | 2.558 | 2.713 | 1.943 |
| Guinea |  | 2.302 | 2.568 | 1.994 |
| Kenya |  | 1.342 | 2.049 | 1.949 |
| Madagascar |  | 1.247 | 1.89 | 1.776 |
| Malawi |  | 2.295 | 2.592 | 2.011 |
| Mali |  | 2.218 | 2.532 | 1.989 |
| Mauritania |  | 1.745 | 2.243 | 1.95 |
| Nigeria |  | 1.173 | 1.885 | 1.815 |
| Senegal |  | 2.131 | 2.476 | 1.994 |
| Tanzania |  | 2.512 | 2.708 | 2.009 |
|  |  |  |  |  |
| Cameroon | *glurp* | -0.446 | 1.071 | 1.845 |
| Congo |  | -0.135 | 1.201 | 1.745 |
| Cote_dIvoire | | -0.181 | 1.234 | 1.803 |
| Ethiopia |  | 1.32 | 1.365 | 1.097 |
| Gabon |  | -0.327 | 1.128 | 1.715 |
| Gambia |  | -0.428 | 1.089 | 1.831 |
| Ghana |  | -0.071 | 1.218 | 1.791 |
| Guinea |  | 0.062 | 1.297 | 1.767 |
| Kenya |  | -0.187 | 1.171 | 1.685 |
| Madagascar |  | -0.177 | 1.178 | 1.596 |
| Malawi |  | 0.046 | 1.263 | 1.741 |
| Mali |  | -0.361 | 1.116 | 1.847 |
| Mauritania |  | -0.383 | 1.118 | 1.778 |
| Nigeria |  | -0.25 | 1.201 | 1.707 |
| Senegal |  | -0.348 | 1.136 | 1.834 |
| Tanzania |  | -0.082 | 1.231 | 1.804 |
|  |  |  |  |  |
| Cameroon | *msp1* | 0.849 | 2.216 | 2.828 |
| Congo |  | 1.14 | 2.253 | 2.486 |
| Cote_dIvoire | | 0.178 | 1.785 | 2.53 |
| Ethiopia |  | 3.033 | 2.892 | 2.063 |
| Gabon |  | -0.288 | 1.53 | 2.462 |
| Gambia |  | 1.463 | 2.461 | 2.558 |
| Ghana |  | 0.604 | 2.124 | 3.065 |
| Guinea |  | 0.432 | 1.994 | 2.842 |
| Kenya |  | -0.148 | 1.599 | 2.468 |
| Madagascar |  | -0.456 | 1.345 | 2.043 |
| Malawi |  | 0.079 | 1.798 | 2.969 |
| Mali |  | 0.204 | 1.871 | 2.936 |
| Mauritania |  | -0.233 | 1.574 | 2.577 |
| Nigeria |  | 0.763 | 1.89 | 2.083 |
| Senegal |  | 0.11 | 1.797 | 2.783 |
| Tanzania |  | 0.179 | 1.862 | 2.988 |
|  |  |  |  |  |
| Cameroon | *phistb* | 0.725 | 1.326 | 1.321 |
| Congo |  | 0.298 | 1.135 | 1.321 |
| Cote_dIvoire | | 0.675 | 1.283 | 1.297 |
| Ethiopia |  | 1.05 | 1.189 | 1.004 |
| Gabon |  | 0.053 | 1.107 | 1.415 |
| Gambia |  | 0.874 | 1.373 | 1.298 |
| Ghana |  | 1.112 | 1.469 | 1.256 |
| Guinea |  | 0.569 | 1.275 | 1.351 |
| Kenya |  | 0.249 | 1.069 | 1.245 |
| Madagascar |  | 0.941 | 1.229 | 1.1 |
| Malawi |  | 0.583 | 1.216 | 1.257 |
| Mali |  | 0.628 | 1.283 | 1.324 |
| Mauritania |  | 0.591 | 1.282 | 1.346 |
| Nigeria |  | 1.404 | 1.512 | 1.227 |
| Senegal |  | 1.047 | 1.455 | 1.307 |
| Tanzania |  | 0.572 | 1.208 | 1.252 |
|  |  |  |  |  |
| Cameroon | *surfin8.2* | 3.484 | 4.012 | 3.271 |
| Congo |  | 2.84 | 3.454 | 2.944 |
| Cote_dIvoire | | 2.811 | 3.289 | 2.69 |
| Ethiopia |  | 2.375 | 2.667 | 2.144 |
| Gabon |  | 2.504 | 3.062 | 2.591 |
| Gambia |  | 3.021 | 3.639 | 3.094 |
| Ghana |  | 4.113 | 4.551 | 3.521 |
| Guinea |  | 3.269 | 3.79 | 3.099 |
| Kenya |  | 2.655 | 3.159 | 2.618 |
| Madagascar |  | 1.937 | 2.471 | 2.144 |
| Malawia |  | 3.378 | 3.972 | 3.323 |
| Mali |  | 3.403 | 3.953 | 3.255 |
| Mauritania |  | 2.649 | 3.244 | 2.762 |
| Nigeria |  | 2.108 | 2.643 | 2.267 |
| Senegal |  | 3.032 | 3.613 | 3.033 |
| Tanzania |  | 3.418 | 4.007 | 3.344 |
|  |  |  |  |  |
| Cameroon | *surfin14.1* | 2.448 | 2.724 | 2.088 |
| Congo |  | 1.836 | 2.391 | 2.095 |
| Cote_dIvoire | | 2.236 | 2.529 | 2.017 |
| Ethiopia |  | 1.582 | 2.023 | 1.769 |
| Gabon |  | 1.437 | 2.157 | 2.041 |
| Gambia |  | 1.899 | 2.423 | 2.087 |
| Ghana |  | 2.426 | 2.769 | 2.128 |
| Guinea |  | 2.28 | 2.607 | 2.065 |
| Kenya |  | 1.986 | 2.394 | 2.002 |
| Madagascar |  | 2.364 | 2.365 | 1.789 |
| Malawi |  | 2.506 | 2.79 | 2.133 |
| Mali |  | 2.433 | 2.698 | 2.065 |
| Mauritania |  | 1.584 | 2.254 | 2.082 |
| Nigeria |  | 1.968 | 2.252 | 1.844 |
| Senegal |  | 1.747 | 2.366 | 2.125 |
| Tanzania |  | 2.909 | 3.01 | 2.132 |

**Supplementary Table 3.** Haplotype frequencies of the vaccine candidates

| **Anigen** | **Haplotype** | **Countries** | **Number** | **Frequency** |
| --- | --- | --- | --- | --- |
| *celtos* | XL | Cameroon | 9 | 0.75 |
|  | VII | Congo | 17 | 0.097 |
|  | VII | Cote_dIvoire | 6 | 0.034 |
|  | LXXXIV | Ethiopia | 10 | 0.256 |
|  | VII | Gabon | 6 | 0.034 |
|  | VII | Gambia | 16 | 0.091 |
|  | VII | Ghana | 36 | 0.205 |
|  | VII | Guinea | 13 | 0.074 |
|  | VII | Kenya | 3 | 0.017 |
|  | CCLIX | Madagascar | 3 | 0.214 |
|  | XI | Malawi | 9 | 0.36 |
|  | XLV | Malawi | 9 | 0.167 |
|  | VII | Mali | 20 | 0.114 |
|  | VII | Mauritania | 7 | 0.04 |
|  | VII | Nigeria | 8 | 0.045 |
|  | VII | Senegal | 14 | 0.08 |
|  | VII | Tanzania | 16 | 0.091 |
|  |  |  |  |  |
| *csp* | I | Cameroon | 32 | 0.087 |
|  | I | Congo | 14 | 0.038 |
|  | I | Cote_dIvoire | 10 | 0.027 |
|  | XCIII | Ethiopia | 21 | 0.84 |
|  | IX | Gabon | 6 | 0.031 |
|  | XXII | Gabon | 6 | 0.031 |
|  | I | Gambia | 23 | 0.063 |
|  | I | Ghana | 80 | 0.218 |
|  | I | Guinea | 26 | 0.071 |
|  | I | Kenya | 10 | 0.027 |
|  | XXII | Madagascar | 4 | 0.02 |
|  | I | Malawi | 46 | 0.125 |
|  | I | Mali | 35 | 0.095 |
|  | I | Mauritania | 15 | 0.041 |
|  | I | Nigeria | 6 | 0.016 |
|  | I | Senegal | 23 | 0.063 |
|  | I | Tanzania | 39 | 0.106 |
|  |  |  |  |  |
| *lsa3* | X | Cameroon | 44 | 0.419 |
|  | III | Congo | 18 | 0.175 |
|  | VIII | Cote_dIvoire | 9 | 0.025 |
|  | CX | Ethiopia | 8 | 0.667 |
|  | III | Gabon | 9 | 0.087 |
|  | VIII | Gambia | 28 | 0.079 |
|  | VIII | Ghana | 86 | 0.242 |
|  | VIII | Guinea | 21 | 0.059 |
|  | XIII | Kenya | 10 | 0.09 |
|  | II | Madagascar | 9 | 0.033 |
|  | VIII | Malawi | 50 | 0.141 |
|  | II | Mali | 29 | 0.108 |
|  | VII | Mali | 29 | 0.115 |
|  | VIII | Mali | 29 | 0.082 |
|  | XI | Mauritania | 10 | 0.079 |
|  | III | Nigeria | 5 | 0.049 |
|  | VIII | Nigeria | 5 | 0.014 |
|  | V | Senegal | 16 | 0.095 |
|  | VIII | Senegal | 16 | 0.045 |
|  | VIII | Tanzania | 47 | 0.132 |
|  |  |  |  |  |
| *trap* | LVIII | Cameroon | 7 | 0.875 |
|  | CCCL | Congo | 2 | 0.2 |
|  | MCCXIII | Congo | 2 | 0.333 |
|  | MCDLXXV | Congo | 2 | 0.4 |
|  | MDCCI | Congo | 2 | 1 |
|  | MDCLXXXIII | Congo | 2 | 1 |
|  | MDCLXXXVI | Congo | 2 | 1 |
|  | MDCXCV | Congo | 2 | 1 |
|  | MDCXCVI | Congo | 2 | 1 |
|  | MDCXXXVIII | Congo | 2 | 1 |
|  | CIII | Cote_dIvoire | 3 | 0.079 |
|  | XLI | Cote_dIvoire | 3 | 0.061 |
|  | MCCCLVIII | Ethiopia | 9 | 1 |
|  | CDXXXVIII | Gabon | 4 | 0.8 |
|  | CIII | Gambia | 10 | 0.263 |
|  | XLI | Gambia | 10 | 0.204 |
|  | XLI | Ghana | 12 | 0.245 |
|  | XXX | Guinea | 4 | 0.667 |
|  | CCCLXXXVI | Kenya | 3 | 1 |
|  | MCXIX | Madagascar | 2 | 0.25 |
|  | CCCXCVIII | Malawi | 4 | 0.364 |
|  | MCXXXV | Malawi | 4 | 0.286 |
|  | XL | Malawi | 4 | 0.121 |
|  | XLI | Mali | 8 | 0.163 |
|  | DLXIV | Mauritania | 5 | 1 |
|  | CIII | Nigeria | 2 | 0.053 |
|  | CIII | Senegal | 11 | 0.289 |
|  | MCXXXV | Tanzania | 10 | 0.714 |
|  |  |  |  |  |
| *glurp* | I | Cameroon | 36 | 0.106 |
|  | VII | Congo | 12 | 0.05 |
|  | VII | Cote_dIvoire | 7 | 0.029 |
|  | XXIX | Ethiopia | 8 | 0.086 |
|  | I | Gabon | 6 | 0.018 |
|  | IV | Gabon | 6 | 0.031 |
|  | IV | Gambia | 26 | 0.136 |
|  | I | Ghana | 74 | 0.217 |
|  | I | Guinea | 30 | 0.088 |
|  | I | Kenya | 14 | 0.041 |
|  | III | Madagascar | 3 | 0.027 |
|  | LXXVII | Madagascar | 3 | 0.2 |
|  | XXVIII | Madagascar | 3 | 0.028 |
|  | I | Malawi | 43 | 0.126 |
|  | I | Mali | 33 | 0.097 |
|  | IV | Mauritania | 9 | 0.047 |
|  | VII | Mauritania | 9 | 0.038 |
|  | I | Nigeria | 4 | 0.012 |
|  | VI | Senegal | 18 | 0.098 |
|  | I | Tanzania | 38 | 0.111 |
|  |  |  |  |  |
| *ama1* | CLXXX | Cameroon | 7 | 0.206 |
|  | CCLXXI | Congo | 6 | 0.545 |
|  | XIII | Cote_dIvoire | 4 | 0.114 |
|  | CDLXXVII | Ethiopia | 7 | 0.778 |
|  | CDXI | Gabon | 3 | 0.6 |
|  | CDXXIV | Gabon | 3 | 0.375 |
|  | CDLIII | Gambia | 4 | 0.667 |
|  | CXVII | Gambia | 4 | 0.167 |
|  | XIII | Ghana | 9 | 0.257 |
|  | XXIX | Guinea | 5 | 1 |
|  | CCCLVI | Kenya | 2 | 1 |
|  | CCCLXIX | Kenya | 2 | 1 |
|  | CCCXCVII | Kenya | 2 | 0.083 |
|  | MCCLIII | Madagascar | 2 | 1 |
|  | MCCXLIII | Madagascar | 2 | 0.667 |
|  | CDXXVII | Malawi | 7 | 0.438 |
|  | CXVII | Mali | 7 | 0.292 |
|  | DXXX | Mauritania | 4 | 0.5 |
|  | XXXIX | Nigeria | 2 | 0.1 |
|  | XIII | Senegal | 10 | 0.286 |
|  | CCCXCVII | Tanzania | 15 | 0.625 |
|  |  |  |  |  |
| *eba175* | CLXX | Cameroon | 9 | 0.281 |
|  | LXXXII | Cameroon | 9 | 0.141 |
|  | XXXI | Cameroon | 9 | 0.084 |
|  | XXXI | Congo | 7 | 0.065 |
|  | CLXX | Cote_dIvoire | 3 | 0.094 |
|  | XIX | Cote_dIvoire | 3 | 0.077 |
|  | XXXI | Cote_dIvoire | 3 | 0.028 |
|  | MXVII | Ethiopia | 6 | 1 |
|  | XLIII | Gabon | 3 | 0.041 |
|  | XXXI | Gabon | 3 | 0.028 |
|  | XXXI | Gambia | 18 | 0.168 |
|  | XLIII | Ghana | 24 | 0.324 |
|  | I | Guinea | 5 | 0.238 |
|  | II | Guinea | 5 | 0.263 |
|  | XIX | Guinea | 5 | 0.128 |
|  | XXVII | Kenya | 3 | 0.107 |
|  | CXIV | Madagascar | 2 | 0.111 |
|  | LXV | Madagascar | 2 | 0.167 |
|  | LXXXII | Malawi | 8 | 0.125 |
|  | XXVII | Malawi | 8 | 0.286 |
|  | XXXI | Mali | 16 | 0.15 |
|  | XIX | Mauritania | 6 | 0.154 |
|  | XXXI | Nigeria | 4 | 0.037 |
|  | XXXI | Senegal | 12 | 0.112 |
|  | LXXXII | Tanzania | 15 | 0.234 |
|  |  |  |  |  |
| *msp1* | CMXXXIX | Cameroon | 4 | 1 |
|  | MMXLII | Congo | 3 | 1 |
|  | CCCXCVII | Cote_dIvoire | 2 | 1 |
|  | CCCXCVIII | Cote_dIvoire | 2 | 1 |
|  | CDIV | Cote_dIvoire | 2 | 1 |
|  | MDCXCV | Ethiopia | 4 | 1 |
|  | DXLIX | Gabon | 2 | 1 |
|  | DXXI | Gabon | 2 | 0.5 |
|  | DXXXVII | Gabon | 2 | 1 |
|  | DLVI | Gambia | 8 | 0.8 |
|  | CCXVI | Ghana | 4 | 1 |
|  | CLIII | Guinea | 2 | 0.667 |
|  | CXLIV | Guinea | 2 | 1 |
|  | CDLI | Kenya | 4 | 0.8 |
|  | MDCCXII | Madagascar | 3 | 1 |
|  | MCCCXCIV | Malawi | 2 | 0.182 |
|  | MCCCXLVIII | Malawi | 2 | 0.667 |
|  | MCCLXXVI | Malawi | 2 | 0.5 |
|  | MCDXCVIII | Malawi | 2 | 0.667 |
|  | MDCCCLXIII | Malawi | 2 | 1 |
|  | MDCCCXCIII | Malawi | 2 | 1 |
|  | MDCCXCVI | Malawi | 2 | 1 |
|  | MMXXI | Malawi | 2 | 1 |
|  | MMXXX | Malawi | 2 | 1 |
|  | CCCX | Mali | 2 | 1 |
|  | CCXLVII | Mali | 2 | 1 |
|  | DCCCLXIII | Mali | 2 | 1 |
|  | DCLXIX | Mauritania | 4 | 1 |
|  | CLXXII | Nigeria | 1 | 0.333 |
|  | CXL | Nigeria | 1 | 0.2 |
|  | MDCCXL | Nigeria | 1 | 1 |
|  | MDCCXLI | Nigeria | 1 | 1 |
|  | MDCCXLII | Nigeria | 1 | 1 |
|  | MDCCXLIII | Nigeria | 1 | 1 |
|  | MDCCXLIV | Nigeria | 1 | 1 |
|  | MDCCXLV | Nigeria | 1 | 1 |
|  | MDCCXLVI | Nigeria | 1 | 1 |
|  | MDCCXXIII | Nigeria | 1 | 1 |
|  | MDCCXXIV | Nigeria | 1 | 1 |
|  | MDCCXXIX | Nigeria | 1 | 1 |
|  | MDCCXXV | Nigeria | 1 | 1 |
|  | MDCCXXVI | Nigeria | 1 | 1 |
|  | MDCCXXVII | Nigeria | 1 | 1 |
|  | MDCCXXVIII | Nigeria | 1 | 1 |
|  | MDCCXXX | Nigeria | 1 | 1 |
|  | MDCCXXXI | Nigeria | 1 | 1 |
|  | MDCCXXXII | Nigeria | 1 | 1 |
|  | MDCCXXXIII | Nigeria | 1 | 1 |
|  | MDCCXXXIV | Nigeria | 1 | 1 |
|  | MDCCXXXIX | Nigeria | 1 | 1 |
|  | MDCCXXXV | Nigeria | 1 | 1 |
|  | MDCCXXXVI | Nigeria | 1 | 1 |
|  | MDCCXXXVII | Nigeria | 1 | 1 |
|  | MDCCXXXVIII | Nigeria | 1 | 1 |
|  | MDCXVIII | Nigeria | 1 | 0.2 |
|  | MMCXL | Nigeria | 1 | 1 |
|  | MMCXLI | Nigeria | 1 | 1 |
|  | MMCXLII | Nigeria | 1 | 1 |
|  | MMCXLIII | Nigeria | 1 | 1 |
|  | MMCLXII | Senegal | 8 | 1 |
|  | MCCCXCIV | Tanzania | 9 | 0.818 |
|  |  |  |  |  |
| *phistb* | II | Cameroon | 61 | 0.093 |
|  | I | Congo | 35 | 0.064 |
|  | II | Cote_dIvoire | 20 | 0.03 |
|  | I | Ethiopia | 12 | 0.022 |
|  | I | Gabon | 17 | 0.031 |
|  | II | Gabon | 17 | 0.026 |
|  | II | Gambia | 44 | 0.067 |
|  | II | Ghana | 141 | 0.214 |
|  | I | Guinea | 35 | 0.064 |
|  | I | Kenya | 25 | 0.046 |
|  | I | Madagascar | 12 | 0.022 |
|  | II | Malawi | 87 | 0.132 |
|  | II | Mali | 51 | 0.077 |
|  | II | Mauritania | 24 | 0.036 |
|  | II | Nigeria | 11 | 0.017 |
|  | II | Senegal | 35 | 0.053 |
|  | II | Tanzania | 86 | 0.131 |
|  |  |  |  |  |
| *surfin8.2* | MLI | Cameroon | 3 | 1 |
|  | MLXXXI | Cameroon | 3 | 1 |
|  | MMCXXX | Congo | 2 | 1 |
|  | MMCXXXIV | Congo | 2 | 1 |
|  | CXCIII | Cote_dIvoire | 3 | 0.75 |
|  | MDCCXIV | Ethiopia | 5 | 1 |
|  | MDCCXVII | Ethiopia | 5 | 1 |
|  | DXXXVIII | Gabon | 3 | 1 |
|  | DLXVII | Gambia | 3 | 1 |
|  | MDCCLXXVII | Gambia | 3 | 1 |
|  | CCXIV | Ghana | 3 | 1 |
|  | DCCLIX | Ghana | 3 | 1 |
|  | DCCLXI | Ghana | 3 | 1 |
|  | DCCLXIV | Ghana | 3 | 1 |
|  | DCCXCVIII | Ghana | 3 | 1 |
|  | DCCXIV | Ghana | 3 | 1 |
|  | DCCXLI | Ghana | 3 | 1 |
|  | DCCXLIX | Ghana | 3 | 1 |
|  | MDCXXV | Ghana | 3 | 1 |
|  | MXCIII | Ghana | 3 | 0.75 |
|  | II | Guinea | 5 | 0.5 |
|  | CDL | Kenya | 1 | 1 |
|  | CDLI | Kenya | 1 | 1 |
|  | CDLII | Kenya | 1 | 1 |
|  | CDLIII | Kenya | 1 | 1 |
|  | CDLIV | Kenya | 1 | 1 |
|  | CDLIX | Kenya | 1 | 1 |
|  | CDLV | Kenya | 1 | 1 |
|  | CDLVI | Kenya | 1 | 1 |
|  | CDLVII | Kenya | 1 | 1 |
|  | CDLVIII | Kenya | 1 | 1 |
|  | CDLX | Kenya | 1 | 1 |
|  | CDLXI | Kenya | 1 | 1 |
|  | CDLXII | Kenya | 1 | 1 |
|  | CDLXIII | Kenya | 1 | 1 |
|  | CDLXIV | Kenya | 1 | 1 |
|  | CDLXIX | Kenya | 1 | 1 |
|  | CDLXV | Kenya | 1 | 0.5 |
|  | CDLXVI | Kenya | 1 | 1 |
|  | CDLXVII | Kenya | 1 | 1 |
|  | CDLXVIII | Kenya | 1 | 1 |
|  | CDLXX | Kenya | 1 | 1 |
|  | CDLXXI | Kenya | 1 | 1 |
|  | CDLXXII | Kenya | 1 | 1 |
|  | CDLXXIII | Kenya | 1 | 1 |
|  | CDLXXIV | Kenya | 1 | 1 |
|  | CDLXXIX | Kenya | 1 | 1 |
|  | CDLXXV | Kenya | 1 | 1 |
|  | CDLXXVI | Kenya | 1 | 1 |
|  | CDLXXVII | Kenya | 1 | 1 |
|  | CDLXXVIII | Kenya | 1 | 1 |
|  | CDLXXX | Kenya | 1 | 1 |
|  | CDLXXXI | Kenya | 1 | 1 |
|  | CDLXXXII | Kenya | 1 | 1 |
|  | CDLXXXIII | Kenya | 1 | 1 |
|  | CDLXXXIV | Kenya | 1 | 1 |
|  | CDLXXXIX | Kenya | 1 | 1 |
|  | CDLXXXV | Kenya | 1 | 1 |
|  | CDLXXXVI | Kenya | 1 | 1 |
|  | CDLXXXVII | Kenya | 1 | 1 |
|  | CDLXXXVIII | Kenya | 1 | 1 |
|  | CDXC | Kenya | 1 | 1 |
|  | CDXCI | Kenya | 1 | 1 |
|  | CDXCII | Kenya | 1 | 1 |
|  | CDXCIII | Kenya | 1 | 1 |
|  | CDXCIV | Kenya | 1 | 1 |
|  | CDXCIX | Kenya | 1 | 1 |
|  | CDXCV | Kenya | 1 | 1 |
|  | CDXCVI | Kenya | 1 | 1 |
|  | CDXCVII | Kenya | 1 | 1 |
|  | CDXCVIII | Kenya | 1 | 1 |
|  | CDXLI | Kenya | 1 | 1 |
|  | CDXLII | Kenya | 1 | 1 |
|  | CDXLIII | Kenya | 1 | 1 |
|  | CDXLIV | Kenya | 1 | 1 |
|  | CDXLIX | Kenya | 1 | 1 |
|  | CDXLV | Kenya | 1 | 1 |
|  | CDXLVI | Kenya | 1 | 1 |
|  | CDXLVII | Kenya | 1 | 1 |
|  | CDXLVIII | Kenya | 1 | 1 |
|  | D | Kenya | 1 | 1 |
|  | II | Kenya | 1 | 0.1 |
|  | XLV | Madagascar | 2 | 0.2 |
|  | II | Malawi | 3 | 0.3 |
|  | CCCLXI | Mali | 2 | 1 |
|  | CCLXXXIX | Mali | 2 | 0.667 |
|  | CXLVII | Mauritania | 2 | 0.167 |
|  | DCLXXI | Mauritania | 2 | 0.667 |
|  | DCII | Nigeria | 1 | 0.333 |
|  | DCXVIII | Nigeria | 1 | 0.5 |
|  | MCXXI | Nigeria | 1 | 0.333 |
|  | MDCCL | Nigeria | 1 | 1 |
|  | MDCCLI | Nigeria | 1 | 1 |
|  | MDCCLII | Nigeria | 1 | 1 |
|  | MDCCLIII | Nigeria | 1 | 1 |
|  | MDCCLIV | Nigeria | 1 | 1 |
|  | MDCCLIX | Nigeria | 1 | 1 |
|  | MDCCLV | Nigeria | 1 | 1 |
|  | MDCCLVI | Nigeria | 1 | 1 |
|  | MDCCLVII | Nigeria | 1 | 1 |
|  | MDCCLVIII | Nigeria | 1 | 1 |
|  | MDCCLX | Nigeria | 1 | 1 |
|  | MDCCLXI | Nigeria | 1 | 1 |
|  | MDCCLXII | Nigeria | 1 | 1 |
|  | MDCCLXIII | Nigeria | 1 | 1 |
|  | MDCCLXIV | Nigeria | 1 | 1 |
|  | MDCCLXV | Nigeria | 1 | 1 |
|  | MDCCLXVI | Nigeria | 1 | 1 |
|  | MDCCXLIV | Nigeria | 1 | 1 |
|  | MDCCXLIX | Nigeria | 1 | 1 |
|  | MDCCXLV | Nigeria | 1 | 1 |
|  | MDCCXLVI | Nigeria | 1 | 1 |
|  | MDCCXLVII | Nigeria | 1 | 1 |
|  | MDCCXLVIII | Nigeria | 1 | 1 |
|  | MMCLXXXII | Nigeria | 1 | 1 |
|  | MMCLXXXIII | Nigeria | 1 | 1 |
|  | MMCLXXXIV | Nigeria | 1 | 1 |
|  | MMCLXXXV | Nigeria | 1 | 1 |
|  | MMCLXXXVI | Nigeria | 1 | 1 |
|  | MMCCVII | Senegal | 9 | 1 |
|  | MCDIV | Tanzania | 7 | 1 |
|  |  |  |  |  |
| *surfin14.1* | XXXIV | Cameroon | 17 | 0.233 |
|  | DCXXIV | Congo | 3 | 0.333 |
|  | XXXIV | Congo | 3 | 0.041 |
|  | LXVII | Cote_dIvoire | 3 | 0.5 |
|  | XXV | Ethiopia | 6 | 0.222 |
|  | XXXIV | Gabon | 8 | 0.11 |
|  | XXXVII | Gambia | 7 | 0.219 |
|  | XXVI | Ghana | 13 | 0.433 |
|  | II | Guinea | 3 | 0.375 |
|  | XX | Guinea | 3 | 0.273 |
|  | XXV | Guinea | 3 | 0.111 |
|  | LXIX | Kenya | 2 | 0.062 |
|  | LVIII | Madagascar | 2 | 0.111 |
|  | XXXIV | Malawi | 8 | 0.11 |
|  | CCVII | Mali | 6 | 0.3 |
|  | XXXIV | Mali | 6 | 0.082 |
|  | CDLXXXVI | Mauritania | 3 | 1 |
|  | LXIX | Mauritania | 3 | 0.094 |
|  | CCCXXXVII | Nigeria | 2 | 0.222 |
|  | CMLXIX | Nigeria | 2 | 0.5 |
|  | XXVIII | Senegal | 7 | 0.184 |
|  | XXXIV | Tanzania | 8 | 0.11 |

h= haplotypes, S= segregating sites

**Supplementary Table 4**

| **Vaccine candidates** | **Predicted B cell epitopes with regions mapping with high Tajima’s D underlined** |
| --- | --- |
| CSP | NELNYDNAGTNL  EMNYYGKQENWYSLKKNSRSLGE |
| TRAP | VGCHPSDGK |
| LSA3 | NYKSNNKTYNENNNEQITTIFNRTNMNPIKKCHMREKIN |
| CELTOS | LPSFENL  AENVKPPKVDPA |
| AMA1 | AYPIDHEGAEPAPQEQNLFSSIEIVERSNYMGNPWTEYMAKYDIEEV  ERRAEVTSNNEVVVKEEYKDEYADIPEHKPTY  NNGPRYCNKDESKRNSM  DGNCEDIPHVNEFPAI |
| EBA175 | DFGGYSTKAE  HGEISEHKIKNFRKKWWNEFREK  SEHKNNINNCKNIPQEE  DPSYTCFRKEAFSSMP  NQQVQETNINDFSEYHEDINDIN |
| PHISTB | NKETRNRRK  NSHKSPMYYAKETFQQLNE |
| MSP1 | KKLYQAQYDLSIYNKQLEEAHNLISVLE  DKINEIKNPPPANSGNTPNTLLDKNKKIEEHEEKIKEIA  GDLMNPHTKEKINEKIITDNKERKIFINNI  LLEDYEKSKKDYEELLEKFYEMKFNNNFNKDVVDKIFSA |
| SURFIN8.2 | ELANNTTNTYEKKCRDFNY  GIFRKGNKLYKSIENKSESNSMKRSSKRSIGSENEQAISDN  NNKVKLKELHTEFHKDEQEILSSQYENIENA  DNENYDNSNYMKNIIEQKMIILKEYYNRKK |
| SURFIN 14.1 | KDNPNYKKRINPSTYS  DVIKSDNHKKECSDLNYELDDVQELFITSDILTIPKYARQ  NFILNLLTMGDSSITSSGESVRYPMEIRIPHNNIRVLPDSVLPKITPINN |

**Supplementary Figure 1.** Map of countries utilized for analyses was extracted from https://mapchart.net


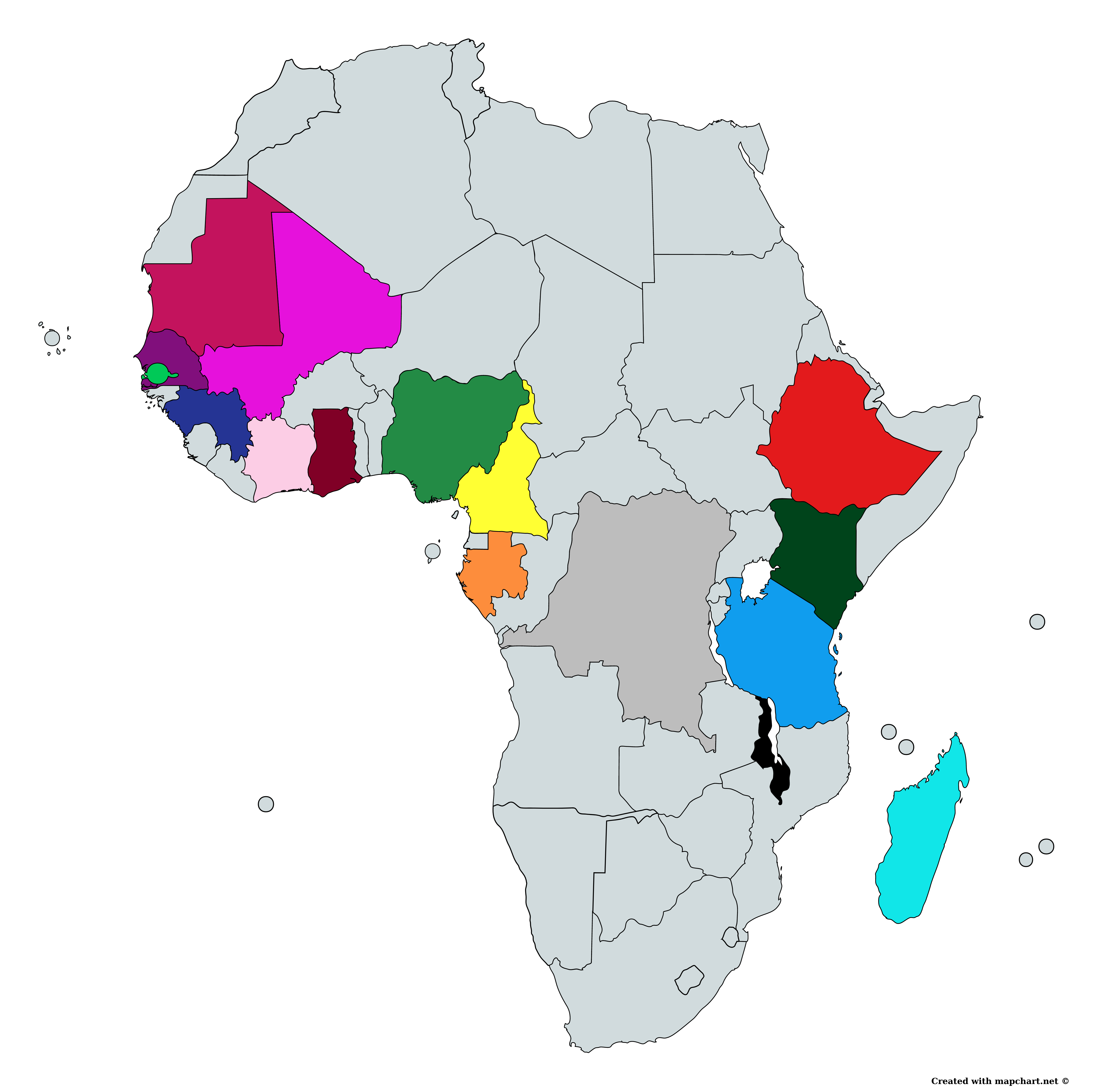

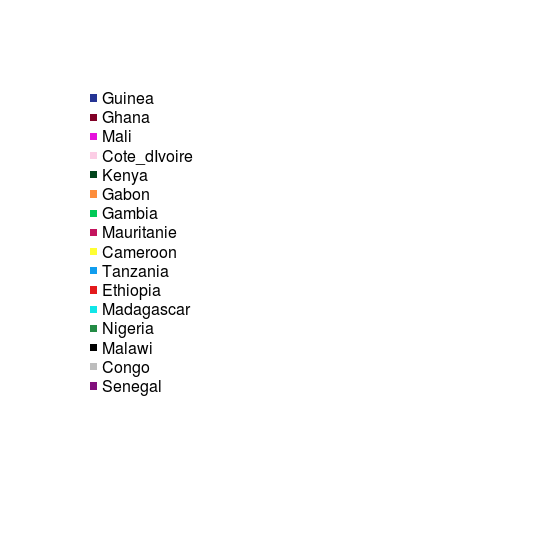


**Supplementary Figure 2.** Sliding window plots for Tajima’s D for (a) pre-erythrocytic antigens (b) erythrocytic antigens. Nucleotide numbers are from the start site of each gene. Window length is 1000 bp.

b.

a.

**Supplementary Figure 3.** Transmembrane helix prediction in Phistb

TMHMM results

PKC46360.1 Length: 485

# PKC46360.1 Number of predicted TMHs: 1

# PKC46360.1 Exp number of AAs in TMHs: 21.37236

# PKC46360.1 Exp number, first 60 AAs: 7.63652

# PKC46360.1 Total prob of N-in: 0.99888

PKC46360.1 TMHMM2.0 inside 1 53

PKC46360.1 TMHMM2.0 TMhelix 54 73

PKC46360.1 TMHMM2.0 outside 74 485

**Supplementary Figure 4.** Posttranslational modification in Phistb


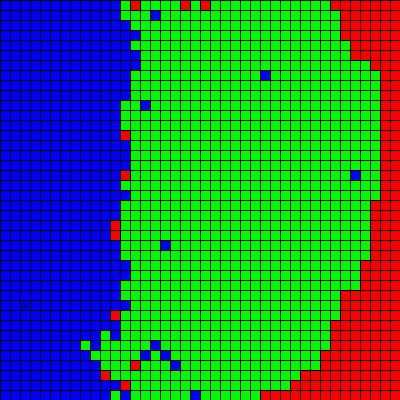


| Seqs submitted: | 1 |
| --- | --- |
| Ignored Seqs (<32AAs): | 0 |
| Seqs with C-terminal signal ([GPI-SOM](http://genomics.unibe.ch/cgi-bin/gpi.cgi?id=63&ch=.log)): | 0 |
| [Undecidable seqs](http://genomics.unibe.ch/cgi-bin/gpi.cgi?id=63&ch=.und): | 0 |
| [**GPI anchored**](http://genomics.unibe.ch/cgi-bin/gpi.cgi?id=63&ch=.pos.gpi) (C&N-term signal) ([SignalP](http://genomics.unibe.ch/cgi-bin/gpi.cgi?id=63&ch=.pos.nt.sig)): |  |
